# The invasive giant African snail *Lissachatina fulica* as natural intermediate host of *Aelurostrongylus abstrusus*, *Angiostrongylus vasorum*, *Troglostrongylus brevior*, and *Crenosoma vulpis* in Colombia

**DOI:** 10.1371/journal.pntd.0007277

**Published:** 2019-04-19

**Authors:** Felipe Penagos-Tabares, Malin K. Lange, Juan Vélez, Jörg Hirzmann, Jesed Gutiérrez-Arboleda, Anja Taubert, Carlos Hermosilla, Jenny J. Chaparro Gutiérrez

**Affiliations:** 1 CIBAV Research Group, Veterinary Medicine School, Faculty of Agrarian Sciences, University of Antioquia, Medellín, Antioquia, Colombia; 2 Institute of Parasitology, Justus Liebig University Giessen, Giessen, Hessen, Germany; University of the District of Columbia, George Washington University School of Medicine and Health Sciences, UNITED STATES

## Abstract

**Background:**

Several metastrongyloid lungworms are unreported pathogens in Colombia. *Angiostrongylus vasorum* and *Crenosoma vulpis* target the cardiopulmonary system of domestic and wild canids. *Aelurostrongylus abstrusus* and *Troglostrongylus brevior* infect felids and considering that six wild felid species exist in Colombia, knowledge of feline lungworm infections is important for their conservation. The zoonotic metastrongyloids *Angiostrongylus costaricensis* and *Angiostrongylus cantonensis* can cause severe gastrointestinal and neurological diseases. *Angiostrongylus costaricensis* has been reported in Colombia, while *Ang*. *cantonensis* is present in neighbouring countries. Research on the epidemiology of metastrongyloids in Colombia and South America more broadly requires evaluating the role that gastropods play as intermediate hosts in their life cycles. This study assessed the prevalence of metastrongyloid larvae in populations of the invasive giant African snail, *Lissachatina fulica*, in Colombia.

**Methodology/Principal findings:**

A total of 609 *Lissachantina fulica* were collected from 6 Colombian municipalities. The snails were then cryo-euthanized, artificially digested and the sediments examined microscopically for the presence of metastrongyloid larvae. Based on morphological characteristics 53.3% (56/107) of the snails from Puerto Leguízamo (Department of Putumayo) were infected with *Ael*. *abstrusus* larvae, 8.4% (9/107) with *Ang*. *vasorum* larvae, 6.5% (7/107) with *T*. *brevior* larvae and 5.6% (6/107) with *C*. *vulpis* larvae, being the region with highest prevalences of the four species. Snails from Andes (Department of Antioquia) and Tulúa (Department of Valle del Cauca) were positive for *Ang*. *vasorum* larvae with a prevalence of 4.6 (11/238) and 6.3% (4/64), respectively. Species identifications were confirmed by PCR and sequencing.

**Conclusions/Significance:**

This epidemiological survey reports for first time the presence of *Ael*. *abstrusus*, *T*. *brevior*, *C*. *vulpis* and *Ang*. *vasorum* in *L*. *fulica* in a number of regions of Colombia.

## Author summary

Several lungworm species are neglected pathogens in Colombia. *Angiostrongylus vasorum* and *Crenosoma vulpis* target the cardiopulmonary system of domestic and wild canids. *Aelurostrongylus abstrusus* and *Troglostrongylus brevior* infect domestic cats as well as wild felids. *Angiostrongylus costaricensis* and *Angiostrongylus cantonensis* may cause severe gastrointestinal or neurological diseases in humans, respectively. Snails/slugs are necessary intermediate hosts in the life cycles of these parasites. We assessed the prevalence of metastrongyloid larvae in 609 specimens of the giant African snail, *Lissachatina fulica*, from 6 Colombian municipalities. In Puerto Leguízamo, 53.3% of the snails were infected with *Ael*. *abstrusus* larvae, 8.4% with *Ang*. *vasorum* larvae, 6.5% with *T*. *brevior* larvae and 5.6% with *C*. *vulpis* larvae. Snails from Andes and Tulúa were positive for *Ang*. *vasorum* larvae with a prevalence of 4.6 and 6.3%, respectively. This epidemiological study reports for first time the presence of *Ael*. *abstrusus*, *T*. *brevior*, *C*. *vulpis* and *Ang*. *vasorum* in the invasive giant African snail in various parts of Colombia.

## Introduction

The giant African snail, *Lissachatina* (= *Achatina*) *fulica*, is originally native to east Africa [1]. It is now one of the most widely distributed and invasive snail species in tropical and subtropical terrestrial ecosystems, and consequently is included among 100 of the world’s worst invasive alien species [2]. As in other South American countries [3], its presence in Colombia has been reported, in 27 of the 32 departments, with the departments of Meta, Valle del Cauca, Putumayo and Caquetá facing a critical ecological threat because of its presence [4]. The giant African snail is a highly invasive alien species in Colombia and is targeted by national campaigns or eradication [4]. Besides the ecological, agricultural and economic threats associated with this introduced snail, it acts as intermediate host of many metastrongyloid nematode species that can cause disease in animals and humans [5–6]. This invasive species constitutes an important intermediate host in the epidemiology of metastrongyloid parasites and contributes to their global dissemination [7–8].

Since 2000 approximately, parasites such as the canine cardio-pulmonary nematode *Angiostrongylus vasorum* and the feline lungworm *Aelurostrongylus abstrusus* have gained increased attention of the veterinary scientific community because of their detection in domestic and wild animals in many countries and their spread into previously non-endemic regions [5,9–12]. Symptoms of canine *Ang*. *vasorum* infections can vary from asymptomatic subclinical infections to cases exhibiting severe cardiopulmonary disorders and coagulopathies that can be fatal [13]. Cases have been reported in Europe, Africa and North and South America [5,9,11,14,15]. It is considered one of the most pathogenic cardiopulmonary nematodes in canids as well as other carnivores [10,16]. Several intermediate gastropod hosts have been reported [17–19], including experimentally infected *L*. *fulica* [20].

*Aelurostrongylus abstrusus* is distributed worldwide and is one of the most important lung parasites in felids [21]. Clinical manifestations of feline aelurostrongylosis are typical of most other respiratory diseases [22]. In addition to domestic cats, *Ael*. *abstrusus* infections are also reported in several wild felid species that may serve as definitive hosts [23,24]. Various species of gastropods have been reported as intermediate hosts [19,25–28].

*Troglostrongylus brevior* is another feline-infecting lungworm that can cause signs ranging from subclinical to severe life-threatening conditions [29]. Its life cycle, symptoms and morphology are very similar to those of *Ael*. *abstrusus*, which has caused confusion in the past [30]. As far as we know, the only intermediate host species reported for *T*. *brevior* is *Cornu aspersum* by experimental infection [31]. There is little information on the biology, epidemiology, pathogenesis and immunology of *T*. *brevior* [32].

*Crenosoma vulpis* is another globally-distributed metastrongyloid, known as the fox lungworm, that invades the bronchi, bronchioles and trachea of wild and domestic canids [33]. Canine crenosomosis is generally characterized by bronchitis with a dry, unproductive cough [34]. However, high parasite burdens can be manifested as a chronic and productive cough [35]. This helminth is endemic in the European and North American red fox (*Vulpes vulpes*) populations [36,37]. In South America *C*. *vulpis* has been recently reported in Chile, where 1% (2/200) of dogs tested were positive for the parasite [38]. Various gastropod species have been reported to be involved in the life cycle of *C*. *vulpis* [19,39].

*Lissachantina fulica* is a known intermediate host of potentially life-threatening human metastrongyloid parasites, the zoonotic species *Angiostrongylus cantonensis* and *Angiostrongylus costaricensis* [40]. In Colombia, *Ang*. *cantonensis* has not been reported, while at least 10 human infections by *Ang*. *costaricensis* have been diagnosed [41]. Neural angiostrongylosis and abdominal angiostrongylosis are infrequently diagnosed because of the poor knowledge of clinicians resulting from the neglected status of these diseases. Nevertheless, in some cases these parasitoses are well tolerated and their subclinical presentation has been described [42, 43].

The parasitic diseases discussed here are considered neglected and their prevalence underestimated in Colombia and South America in general, and therefore further research evaluating their epidemiological status in the region is urgently needed, particularly given the increasing spread of *L*. *fulica*. Thus, the aim of the present study was to determine the epidemiological status of metastrongyloid parasites in *L*. *fulica* populations from the Andean, Pacific and Amazonian Colombian biogeographic regions.

## Material and methods

### Ethics statement

This study was approved on February 2, 2016 by the ethics committee for animal experimentation of the University of Antioquia, Medellín, Colombia, order N° 101. The giant African snail is not among the specially protected fauna regulated by the Act on Nature Conservation and Landscape Management of Colombia.

### Study areas and snail collection

**Fig 1:**
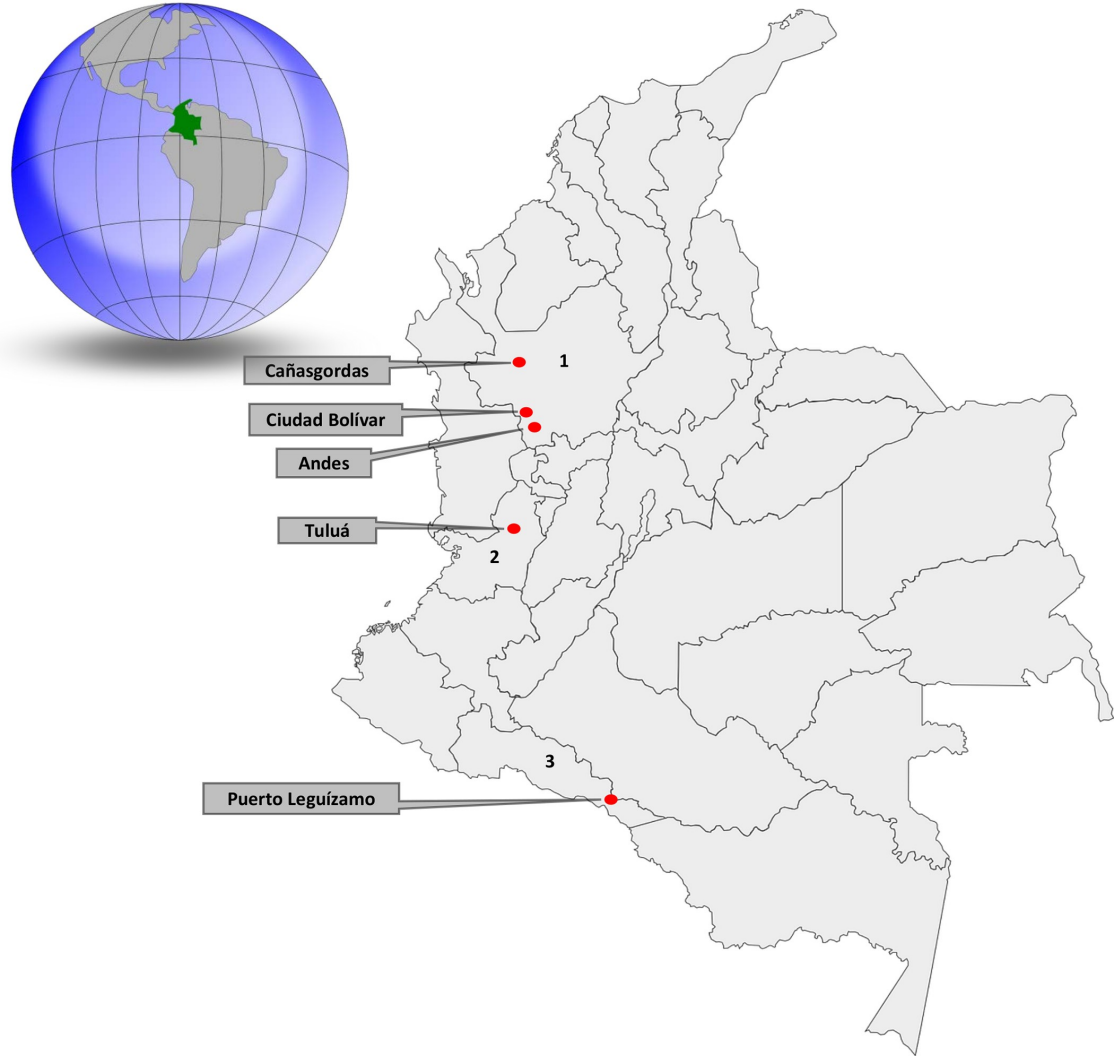
Sampling locations of giant African snails *Lissachatina fulica* in Colombia. Cañasgordas, Ciudad Bolívar and Andes (1.Department of Antioquia) are in the Andean region. Tuluá (2.Department Valle del Cauca) is in the Pacific region and Puerto Leguízamo (3.Department of Putumayo) is in the Amazonian region. This figure has public domain images. (Sources: https://pixabay.com/vectors/colombia-map-geography-36572/ and https://pixabay.com/vectors/colombia-earth-globe-world-153342/).

In total, 609 *L*. *fulica* were collected between February and October of 2016. Of these, 438 were from the Andean region municipalities of: Andes (5° 39′ 20″ N, 75° 52′ 49″ W) (*n* = 238), Ciudad Bolívar (5° 50′ 58″ N, 76° 1′ 13″ W) (*n* = 100), and Cañasgordas (6° 44′ 59″ N, 76° 1′ 33″ W) (*n* = 100), located in the Department of Antioquia. Snails from the Pacific region were collected in the town of Tuluá, Department of Valle del Cauca (4° 5′ 5″ N, 76° 11′ 55″ W) (*n* = 64), and the Amazonian region of Puerto Leguízamo, Department of Putumayo (0° 11′ 38″ S, 74° 46′ 50″ W) (*n* = 107). The specific locations within the Colombian map are shown in Fig 1. The snails were frozen and sent to the parasitology laboratory of the Veterinary Medicine School at the University of Antioquia in Medellín.

### Processing of samples

The snails were morphologically identified, weighed, cut into small pieces and immersed in a digestion solution (10 g pepsin powder 2000 FIP-U/g, 8.5 g NaCl, 30 mL HCl 37% and distilled water to complete 1 L of solution) overnight at 40°C in 50 mL Falcon tubes under constant shaking. Then, the samples were sieved through a 300 μm-metal sieve to remove any undigested material and further passed through a 25 μm-metal sieve. The remnants retained in the last sieving were transferred to 15 mL Falcon tubes and centrifuged at 400 g for 10 min. The pellets were re-suspended and examined microscopically with a light microscope at 4x, 20x and 40x magnification. Metastrongyloid larvae were counted and collected by pipetting. In cases of high larval burden (more than 50 larvae per snail) only 10% of the larvae were viewed at higher magnifications.

### Morphological identification of metastrongyloid larvae

**Fig 2:**
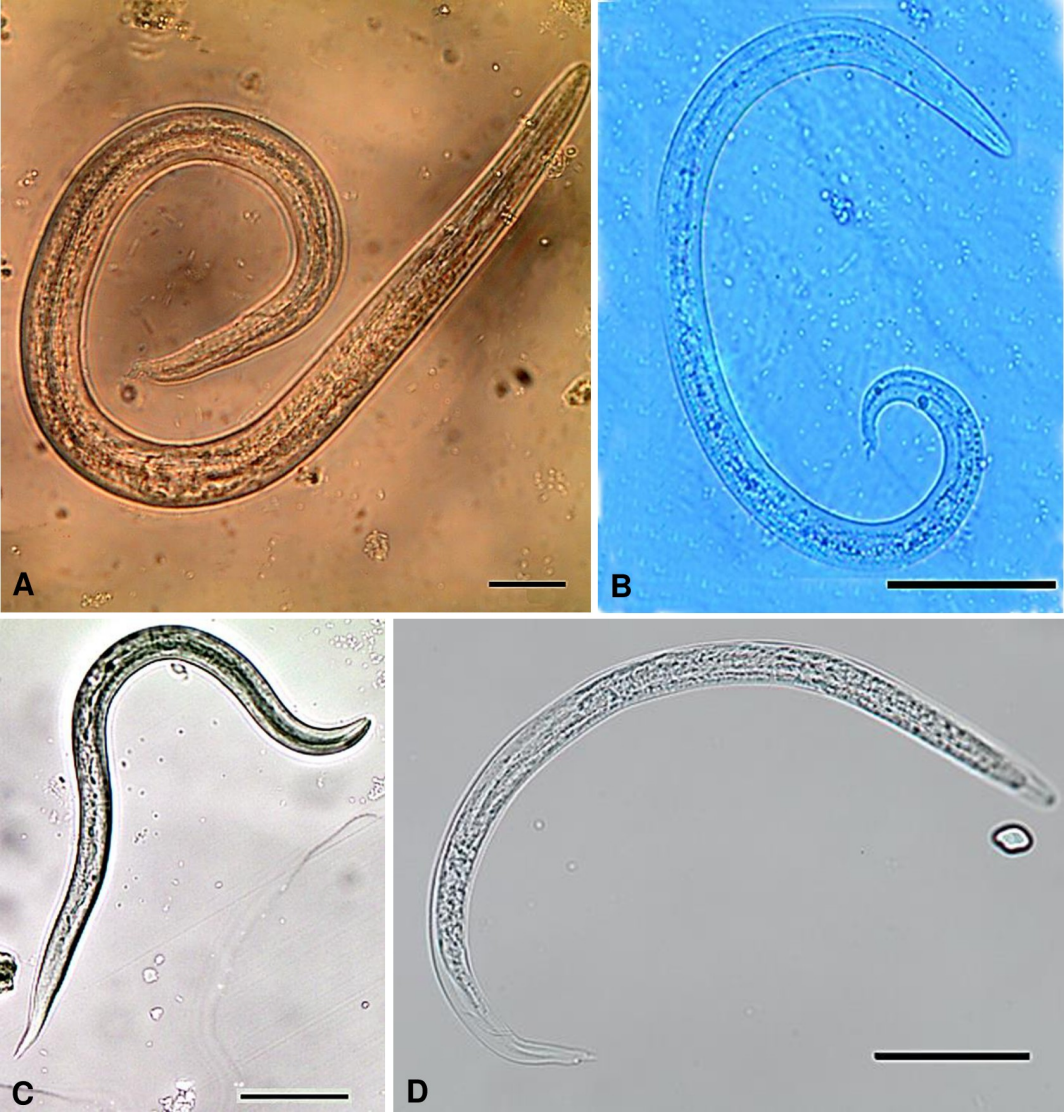
Metastrongyloid larvae found in *Lissachatina fulica* from different regions of Colombia. (A) *Aelurostrongylus abstrusus* L1, scale bar 20 μm. (B) *Troglostrongylus brevior* L1, scale bar 50 μm. (C) *Crenosoma vulpis* L1, scale bar 50 μm. (D) *Angiostrongylus vasorum* L1, scale bar 50 μm.

Larval stages of metastrongyloids were identified by means of body measurement (length/width ratio) and the form (non-rhabditiform) and ratio of oesophagus to body lengths (1:3–1:2) in comparison to other nematodes [10] following Lange et al. 2018 [20]. To distinguish between different metastrongyloid larval stages, the distinct tail morphology of each genus was examined. For illustration of L1, L2 and L3 of *Ang*. *vasorum*, *Ael*. *abstrusus* and *Crenosoma* sp. please see Lange et al. 2018, Fig 2 [20] and regarding L1 see Fig 2. The lungworm species were identified by typical morphometric characteristics [5,9,19,31,39,44–48]. One general feature of metastrongyloid larvae is the non-rhabditiform oesophagus, which forms 1 ⁄ 3–1 ⁄ 2 of the total larval length [9]. *Angiostrongylus vasorum* (310–400 μm × 14–16 μm) is characterized by a small cup as a cephalic button which emerges on the oral extremity and its tail tip with a dorsal spine and sinus wave curve [5,36,43,44,48]. *Aulerostrongylus abstrusus* (300–415 μm × 18–19 μm) has a slender anterior extremity with a short/terminal oral opening leading into a narrow vestibule and its tail is S-shaped with visible dorsal kink, distinct deep dorsal, ventral, incisures, a terminal knob-like extremity [5,19,35,45,48]. *Troglostrongylus brevior* (300–357 μm × 16–19 μm) has a clear and pointed anterior extremity with a sub-terminal oral opening and its tail gradually tapered to dorsal incision, dividing the extremity into two appendices (shallow ventral one, slender dorsal one) S-shaped tail is not as obvious as in *Ael*. *abstrusus*, ending straight, gradually tapered [5,31,46,47]. *Crenosoma vulpis* (240–310 μm × 13 μm) has a pointed and straight tail without indentations and entirely pointed [5,19,39]. The lengths of metastrongyloid larvae vary strongly depending on lungworm species, developmental stage and size of the respective intermediate host [9,44].and were thus not considered as reliable inter-species differentiation feature.

### Confirmation of metastrongyloid larvae via PCR and sequencing

The previously described morphological tail characteristics allowed larvae identification to the genus level. Therefore, for these larvae, those of each genus from a single snail were pooled and underwent additional PCR analyses to identify them at species level. After digestion with proteinase K, DNA from the pooled larvae was isolated using the Qiagen DNeasy Blood and Tissue Kit according to the manufacturer’s protocol, with a final elution volume of 50 μL. To enhance the sensitivity of the molecular diagnosis, nested PCRs were performed. Following a conventional PCR with the universal nematode specific primers NC1/NC2 [49] specific real-time PCR analyses for individual species were performed as described in the literature [19]. Molecular confirmation was attained with the duplex-real-time PCR for *Ael*. *abstrusus* and *T*. *brevior* with melt analysis was carried out, amplifying the internal transcribed spacer 2 (ITS-2) region from the ribosomal DNA (rDNA) of 220 bp (*Ael*. *abstrusus*) and 370 bp (*T*. *brevior*). This PCR was conducted using the forward primers TrogloF, AeluroF and the single reverse primer MetR [50]. For confirm the infections by *Ang*. *vasorum* and *C*. *vulpis* a probe-based duplex-real-time PCR was performed amplifying a partial ITS-2 region as reported by Jefferies et al. 2011 [51]. To account for the inhibitory effects deriving from snail tissue [18, 52], samples were diluted 10 fold with sterile water and tested again with the above PCR conditions. In those cases in which the real-time PCRs were negative or inconclusive and the quantity of the amplicon-DNA from the first PCR was low we performed a second nested conventional PCR with primers NC1/MetR followed by direct sequencing or sequencing after cloning. In addition, we also sequenced some of the samples identified by real-time PCR for confirmation. Hence, 21 samples were purified and sent to a commercial sequencing service (LGC Genomics, Berlin, Germany). The sequences obtained were verified by eye with the software Chromas Lite (version 2.01) and the TCG microsatellite triplet repeat (as part of the ITS-2 region) and nucleotide polymorphisms were used to discriminate between the different reported genotypes by comparing sequences in GenBank via the BLAST algorithm (http://www.ncbi.nlm.nih.gov/BLAST/). The statistical analysis (*p*-value and Pearson’s correlation coefficient) were carried out using Free Statistics Software (v1.2.1), Office for Research Development and Education (https://www.wessa.net/rwasp_correlation.wasp/). The value was considerate significant if *p* < 0.05.

## Results

**Fig 3:**
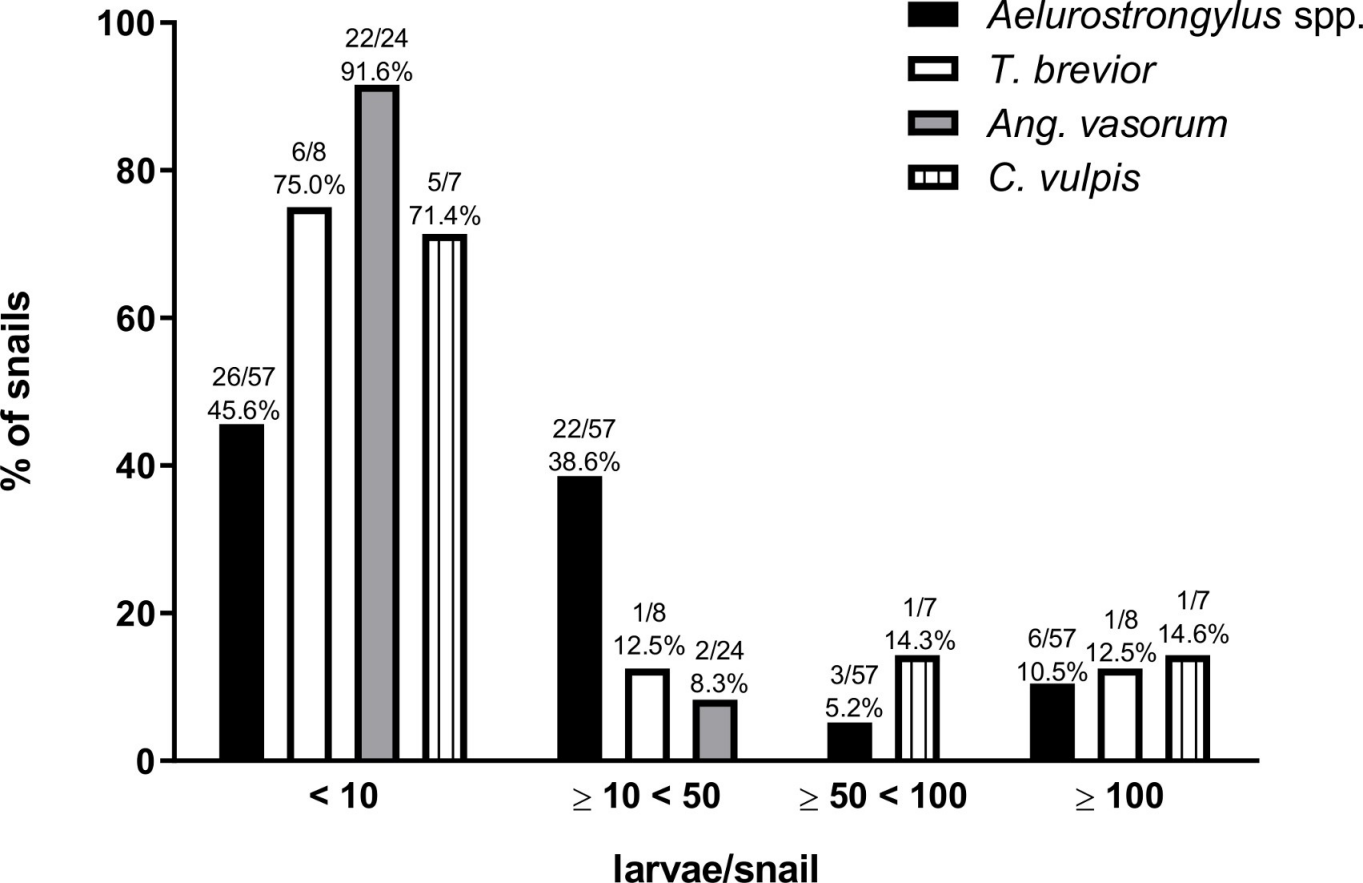
Larval burden categories for *Aelurostrongylus abstrusus*, *Angiostrongylus vasorum*, *Crenosoma vulpis*, *Troglostrongylus brevior* and unknown *Aelurostrongylus* sp. per infection. In this graphic are referred 96 infections, 2 infections of unknown *Angiostrongylus* spp. were not included. Species identification was confirmed via PCR and corroborate by sequencing.

**Fig 4:**
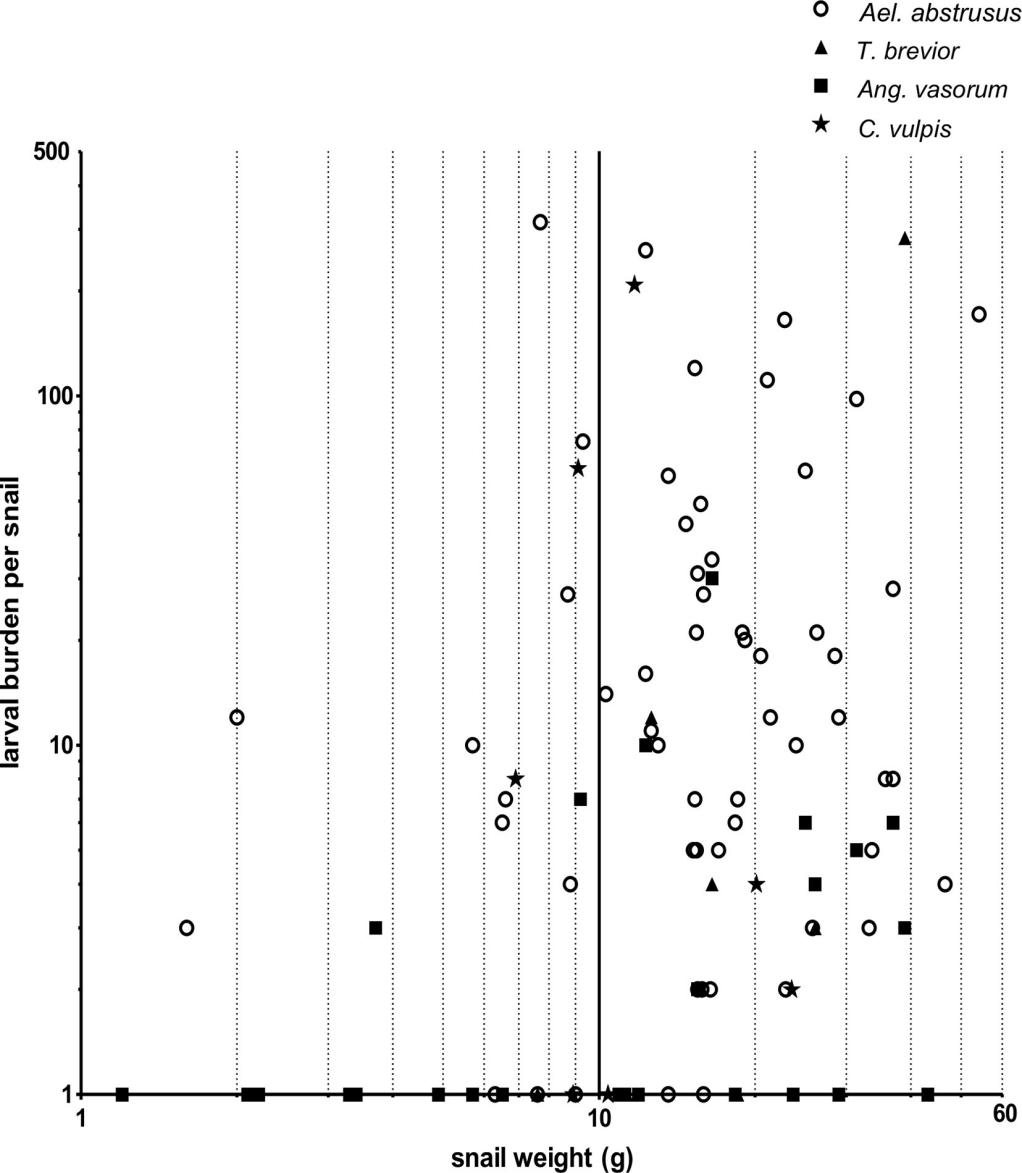
Relationship between (log^10^ axes) snail weight and *Aelurostrongylus* spp., *Angiostrongylus vasorum*, *Crenosoma vulpis* and *Troglostrongylus brevior* larval burden. Here, the larval burden was plotted as a function of the snail weight. *P*-value: 0.065 and Pearson Correlation coefficient (r-value): 0.167. *Aelurostrongylus* includes genotypes A, AB and B of *Aelurostrongylus abstrusus* as well as one unknown *Aelurostrongylus* sp.

**Fig 5:**
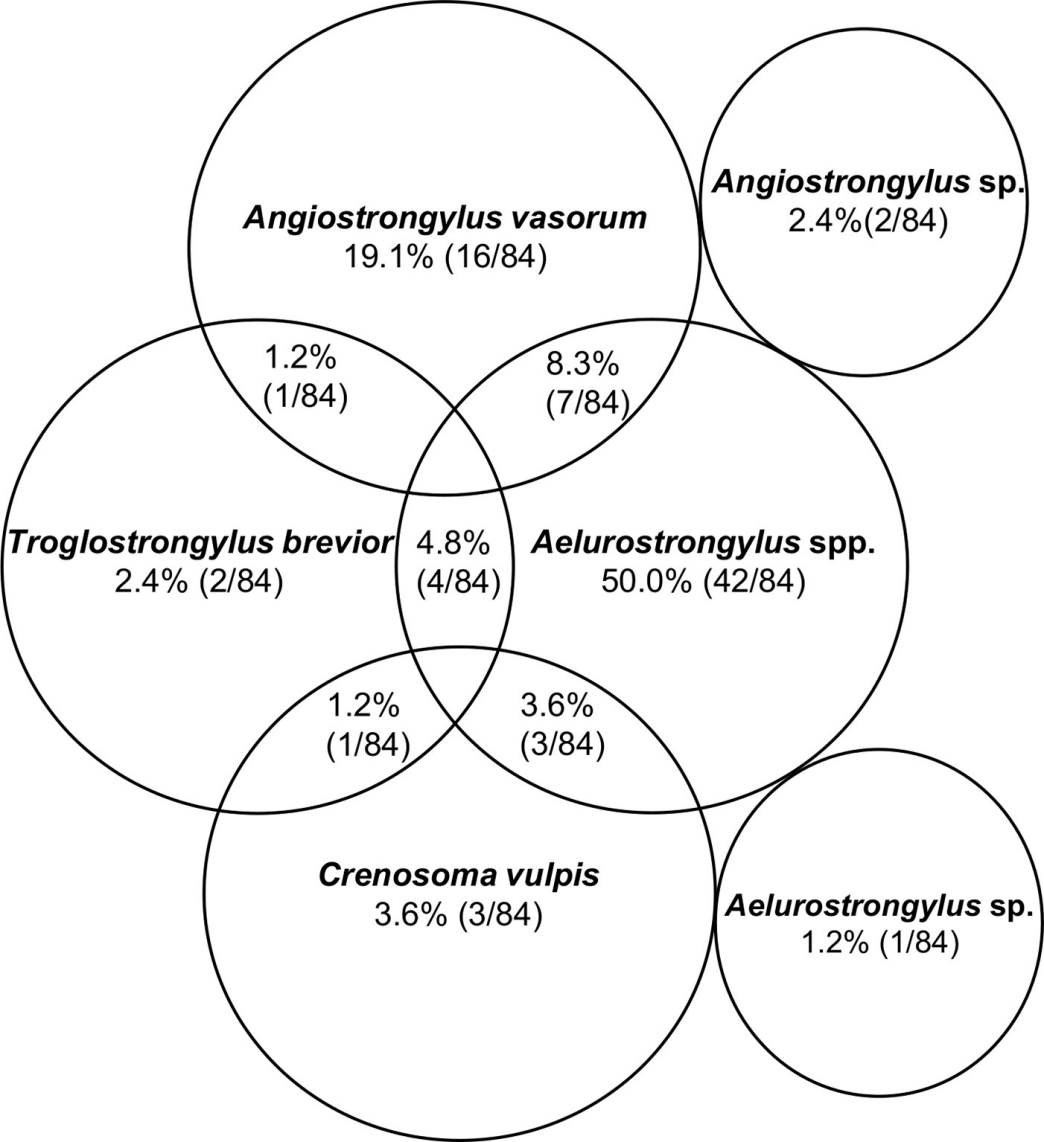
Mono- and co-infections with *Aelurostrongylus abstrusus*, *Angiostrongylus vasorum*, *Crenosoma vulpis* and *Troglostrongylus brevior*, unknown *Aelurostrongylus* sp. and *Angiostrongylus* sp.

In the 609 processed samples, metastrongyloid larvae were identified morphologically, and were found to belong to the following genera: *Aelurostronglyus*, *Troglostrongylus*, *Crenosoma* and *Angiostrongylus* (Fig 2). Molecular analyses with PCR allowed the identification of each lungworm species and overall prevalence in snails from each Colombian region is shown in Table 1. The distinction between different *Angiostrongylus* species was difficult by means of microscopy. Several of the *Angiostrongylus* spp. positive samples contained larvae that resembled *Ang*. *vasorum* (*n* = 24) and some showed characteristics of *Ang*. *cantonensis* (*n* = 2). However, larvae identified as probable *A*. *cantonensis* were not molecularly confirmed because DNA could not be amplified. For larvae of *Ael*. *abstrusus*, *T*. *brevior*, *Ang*. *vasorum* and *C*. *vulpis* morphological identification was confirmed by PCR and sequencing. Molecular biological analyses revealed total prevalences that varied with location and species (Table 1). Larval burden ranged from 1 to 314 larvae per snail for *Ael*. *abstrusus* and from 1 to 286 larvae per snail for *T*. *brevior* (Figs 3 and 4; to see larval burden of each region see S1 Fig). Larval burden for *C*. *vulpis* ranged from 1 to 208 larvae per snail. No relationships between the larval burden and the snail weight were found (Figs 3 and 4). Co-infections involving two species were detected by means of microscopy and PCR in 19.1% (16/84) of all lungworm positive snails (Fig 5). Co-infections consisting of more than two species were not detected.

**Table 1:** Prevalence of metastrongyloid lungworm larvae in *Lissachatina fulica* from 5 geographic regions of Colombia.

Lungworm species detected	Location (number of *L*. *fulica* collected)					Total
	Antioquia			Valle del Cauca	Putumayo	(*n* = 609)
	Andes (*n* = 238)	Cañasgordas (*n* = 100)	Ciudad Bolívar (*n* = 100)	Tuluá (*n* = 64)	Puerto Leguízamo (*n* = 107)	
***Aelurostrongylus abstrusus***	n.d.	n.d.	n.d.	n.d.	52.3% (56)	9.2% (56)
**unknown *Aelurostrongylus* sp.**	n.d	n.d	n.d	n.d	0.9% (1)	0.2% (1)
***Troglostrongylus brevior***	n.d.	n.d.	1.0% (1)	n.d.	6.5% (7)	1.3% (8)
***Crenosoma*** ***Vulpis***	n.d.	1.0% (1)	n.d.	n.d.	5.6% (6)	1.1% (7)
***Angiostrongylus vasorum***	4.6% (11)	n.d.	n.d.	6.3% (4)	8.4% (9)	3.9% (24)
**Unknown *Angiostrongylus* spp.**^+^	0.4% (1)	n.d.	n.d.	1.6% (1)	n.d.	0.3% (2)
**Total**	5.0% (12)	1.0% (1)	1.0% (1)	7.8% (5)	73.8% (79)	16% (98*)

n.d. no larvae detected

^+^These larvae could not be identified by PCR or sequencing. That unspecific identification was based on morphology.

*98 infections by metastrongyloid parasites were detected in 84 snails, 16 of them presented co-infections. See Fig 5.

From the 84 snails positive for metastrongyloid larvae by microscopy, 29 yielded sufficient DNA from larvae pools for sequence analysis, and 25 of those 29 were selected for identification or confirmation by sequencing. Specimens of *Ael*. *abstrusus*, *Ang*. *vasorum*, *C*. *vulpis* and *T*. *brevior* could be confirmed via sequencing with an identity of 99–100% to known GenBank entries (Table 2). Regarding samples positive for *Ael*. *abstrusus* in PCR, sequencing revealed genotype variation with similarities to European genotypes of *Ael abstrsus* of 99% (genotype A), 94% (genotype AB) and 92% (genotype B, Table 2). *Aelurostrongylus abstrusus* genotype A isolates (*n* = 11) contained a microsatellite sequence with 7–10 times TCG from ITS2 sequence. In contrast to genotype B (*n* = 3), which among other nucleotide variations, contained 4 TCG repeat units in the microsatellite and has never been reported before. Genotype AB showed an intermediate number of TCG repeats of 15 (see S1 Table). In addition to these four species one sample contained a sequence for which no match was found in GenBank. It showed 82% similarity to European *Ael*. *abstrusus* isolates, thus probably belonging to the genus of *Aelurostrongylus* (Table 2).

**Table 2:** Identification of metastrongyloids from Colombian giant African snails *Lissachatina fulica* by BLAST search of their ITS2 sequences.

Lungworm species	Accession number	*n*	BLAST search result (program: discontiguous megaBLAST)		
			Accession number	Query cover	Identity^+^
*Aelurostrongylus abstrusus* (A)	MH779453	11	DQ372965*	100%	99%
*Aelurostrongylus abstrusus* (B)	MH779464	3	KM506760*	100%	92%
*Aelurostrongylus abstrusus* (AB)	MH779461	1	KM506760*	100%	94%
Aelurostrongylus sp. (C)	MH780915	1	KM506760*	100%	82%
*Angiostrongylus vasorum*	MG252606	1	GU045371	100%	99%
*Crenosoma vulpis*	MH780053	2	KF836608	100%	100%
*Troglostrongylus brevior*	MH780056	6	KF241978	100%	100%

* *Ael*. *abstrusus* (European genotypes)

^+^it refers similarity to European genotypes. For more details, see S2 Fig, S3 Fig, S4 Fig, S1 and S2 Tables

(A) *Ael*. *abstrusus genotype A*, 99% of similarity to reported European genotypes

(B) *Ael*. *abstrusus* genotype B, 92% of similarity to reported European genotypes

(AB) *Ael*. *abstrusus* genotype AB, 94% of similarity to reported European genotypes

(C) unknown sp of the genus *Aelurostrongylus*, 82% of similarity to reported European genotypes of *Ael*. *Abstrusus*

## Discussion

This survey was aimed at assessing the prevalences of metastrongyloid lungworm species in giant African snail populations from five municipalities of Colombia and adding novel data on the presence of lungworms of domestic animals and wildlife.

The existence of the feline lungworm *Ael*. *abstrusus* in the definitive host population in Colombia, namely domestic cats, has been known since 2003 but has been reported only rarely since [53–55]. The high prevalence detected indicates that Putumayo is a hotspot for this parasite. Large areas of tropical rainforest of Colombia form the natural habitat of six wild felid species: ocelots (*Leopardus pardalis*), oncillas (*Leopardus tigrinus*), margay wildcats (*Leopardus wiedii*), cougars (*Puma concolor*), Jaguarundies (*Puma* (junior synonym: *Herpailurus*) *yagouaroundi*) and Jaguars (*Panthera onca*) [56], populations of which are decreasing and are in greater or lesser degree of threat [57,58]. All these wild felid species could be affected by high prevalences of *Aelurostrongylus* spp., especially considering the high pathogenicity and mortality associated with aelurostrongylosis [21]. Thus, new knowledge on the epidemiological status of lungworm species in wild cat populations from South America is needed to strengthen and consolidate more successful conservation programmes [5]. Additionally, the two different *Aelurostrongylus* genotypes (A and AB) here detected indicate that there is probably more genetic variation in this genus in South America, a region where limited molecular studies have been carried out [5], without reports of other taxa of the genus. This is in line with a report on genetic variability of *Ang*. *vasorum* isolates from South America and Europe [52]., Those three discovered genotypes (A, B, AB) showed different similarity to sequences originating from Europe published in GenBank. Genotype A (99% similarity) can be considered as identical to European isolates. Genotype B, however, shows only 92% similarity to European isolates. With differences of more than 5% a cryptic species or a subspecies could be hypothesised. However, we only analysed larvae, not adults, and for such a hypothesis more analyses would be necessary. Therefore, we here call these different isolates genotypes of *Ael*. *abstrusus*. The genotype AB shows similarities to both genotype A and B (see S2 Fig, S3 Fig, S4 Fig, S1 and S2 Tables). Whether or not genotype AB may represent a hybrid between the other two genotypes is to be answered in the future, conducting in-depth genetic analyses with a larger sample size and also involving isolates from final hosts. Overall, our sequence analysis of ITS2 provides evidence that either *Ael*. *abstrusus* is a species complex comprised of three distinct genotypes (A, B, AB) which may be cryptic species or that it is possible that the genetic variants could represent different *Aelurostrongylus* species. Similar observations exist for several parasites [59]. As for the unknown species which is probably belonging to genus *Aelurostrongylus* (genotype C, 82% similarity to the European genotypes of *Ael*. *abstrusus*, Table 2, S2 Fig), it cannot be yet stated that this is an undiscovered species. It could also represent an already known species for which no sequence was published in GenBank, yet. This should be considered in future studies of lungworms from wild felids in Colombia. and also in other South American countries, where research on lungworms is missing [5].

*Angiostrongylus vasorum* was reported in two Colombian crab-eating foxes (*Cerdocyon thous*) in 1961 and 2014 [60,61], but not yet in the Colombian domestic dog population. Few data exist on the prevalence of *Ang*. *vasorum* in intermediate hosts in other countries and these data vary considerately (1.6–43% prevalence in slug populations) depending on the sampling areas [7,18,19,52,62]. These reports correspond well to the observed rather low *Ang*. *vasorum* prevalence in this survey. Since *Ang*. *vasorum* displays a patchy distribution pattern with hyperendemic foci being in close proximity to areas of low prevalence [36,62,63], further extended epidemiological surveys in Colombia are required to detect hotspots of this canine angiostrongylosis. Surprisingly, recent results of the sequencing of samples positive for *Ang*. *vasorum* from Colombia [64] revealed identity with the European strain and not with the South American strain, which has been suggested as being another species, *Ang*. *raillieti* [65]. As for the other two lungworm species, *C*. *vulpis* and *T*. *brevior*, no reports in Colombia existed prior to our study.

Considering larval burdens, the majority of snails carried fewer than 50 larvae for each of the four found lungworm species (Fig 3). Similar findings have been described before for *Ang*. *vasorum* in natural slug populations [19]. Snails carrying a high larval burden (more than 50 larvae) can be considered most dangerous for the definitive host population since a higher infection dose leads to more severe clinical manifestations [66,67]. These low loads observed in *Ang*. *vasorum* are in line with previous reports in the majority of gastropod species investigated [18,19]. For instance, in Denmark and Germany the percentage of slugs harbouring more than 100 larvae was 14% [17] and 3.3% [19], respectively. Similar findings were also reported for the closely related species *Ang*. *costaricensis* in which 82% of the slugs were infected with low loads [68]. In the present survey, burdens of 100 or more larvae per snail were observed in only 14.3% and 12.5% of the snails regarding *C*. *vulpis*, and *T*. *brevior*, respectively and only 10.5% concerning *Ael*. *abstrusus*, which is in line with another study [19].The observation that the majority of gastropods contain a low metastrongyloid larval burden, with a small minority harbouring high burdens has been described as overdispersion [69], that may lead to subclinical infections, which are frequently observed in the definitive hosts of the four parasite species [21,29,34,70]. It is possible to speculate, therefore, that low larval burdens in gastropods may relate to their innate immune system, with effective formation of so-called invertebrate extracellular phagocyte traps (InEPTs) by gastropod haemocytes in response to metastrongyloid lungworm larvae, which has recently been shown [71]. This question requires, however, further in-depth investigation of how gastropods defend themselves against invading metastrongyloid parasites [72]. The maximal metastrongyloid larval burden found in this survey was 314 *Ael*. *abstrusus* larvae (Fig 3), which is slightly lower than the 392 *Ang*. *vasorum* burden found in a Danish slug [17] and much lower than the 546 *Ang*. *vasorum* larvae found in a German slug [19]. The majority of lungworm positive snails were larger (over 10 g) and probably therefore older. Larger and older snails/slugs have in general more environmental exposure to larvae excreted in faeces, which makes more likely their contact and infection with *Ang*. *cantonensis* and *Ang*. *vasorum* [19,73,74]. Co-infections in the definitive hosts are frequently reported for the two cat lungworm species [75] and the two dog lungworm species [76]. Conversely, in the intermediate hosts, reports of metastrongyloid co-infections are rather scarce. To the best of our knowledge, there has been only one report of multiple simultaneous infections of *Ang*. *vasorum*, *C*. *vulpis* and *Ael*. *abstrusus* in slugs, in Germany [19]. The survey conducted here reports mixed infections of *T*. *brevior* and *Ael*. *abstrusus*, *T*. *brevior* and *C*. *vulpis*, and *T*. *brevior* and *Ang*. *vasorum* for the first time in gastropod hosts (Fig 5), although these co-infections were only occasionally detected.

In addition to *Ang*. *vasorum*, other lungworm larvae of the genus *Angiostrongylus* that resembled *Ang*. *cantonensis* were detected in two regions of Colombia (Table 1) via microscopy. This parasite has never been reported in Colombia, but is present Ecuador, Brazil and the Caribbean islands [77]. In recent decades, several cases of human meningoencephalitis of unknown aetiology have been reported in Colombia [78], which might correspond to cases of human angiostrongyliasis that were unreported or unrecognized [77]. Further specific investigations on this zoonosis are necessary to confirm whether or not it is already circulating in definitive and intermediate hosts as well as in exposed human populations, in order to take measures to inform the Colombian public health institutions and protect society from this life-threatening parasitosis. Although other metastrongyloid nematodes of wildlife were not detected in the snails, it cannot be excluded that they may have been overlooked in co-infections in some samples because of possible DNA degradation.

### Conclusion

To the best of our knowledge, this is the first large-scale survey confirming by molecular analysis the presence of *Ael*. *abstrusus*, *Ang*. *vasorum*, *T*. *brevior* and *C*. *vulpis* infections in intermediate hosts in Colombia. The records of *T*. *brevior* and *C*. *vulpis* represent the first report of these parasites in this country and the first confirmation via molecular techniques of these parasites in South America, demonstrating both 100% similarity to the European genotypes. Interestingly, a hotspot of *Ael*. *abstrusus* (with high genetic variability) and 1 potentially undescribed nematode which could belong to the genus *Aelurostrongylus* were here reported in the Amazonian region, specifically in Putumayo. On the basis of records of *Ang*. *cantonensis* in neighbouring countries and previous reports in Colombia of *Ang*. *costaricensis*, further research on *L*. *Fulica* as well as other natural populations of gastropods such as veronicellid slugs and *Cornu aspersum* should be undertaken in Colombia. The biology of invasive species in the region and their interactions with the native fauna requires more attention and investigation by the national authorities. Thus, more epidemiological and basic research on all these parasites in natural populations of paratenic hosts (such as birds, amphibians, crabs, amongst others) and intermediate hosts in other geographic areas is needed. In the same way data regarding prevalence in humans, domestic animals and wild definitive hosts are required in Colombia as well as in other countries of South, Central and North America to increase knowledge of the impact, dynamics, genetic variation and environmental factors associated with these neglected parasitoses.

## Supporting information

S1 Fig Relationship between (log10 axes) snail weight and *Aelurostrongylus* spp., *Angiostrongylus vasorum*, *Crenosoma vulpis* and *Troglostrongylus brevior* larval burden. A) Larval burden per snail and snail weight of all 609 snails and those collected in B) Puerto Leguízamo (*n* = 107), C) Andes (*n* = 238), D) Tuluá (*n* = 64), E) Cañasgordas (*n* = 100) and F) and Ciudad Bolívar (*n* = 100).
(PDF)

S2 Fig **Alignment CO *Aelurostrongylus* genotypes A, AB, B and C.** Alignment of ITS2 sequences from *Ael. abstrusus* isolates detected during the present study in *Lisachatina fulica* in Colombia (CO). Genotype A is found worldwide and is identical with European isolates of *Ael. abstrusus*, whereas genotypes AB, B, and unknown species C were so far only described from Colombia.
(PDF)

S3 Fig Multiple sequence alignment of *Aelurostrongylus* ITS2 sequences. Alignment of ITS2 sequences from *Ael*. *abstrusus* isolates detected in Colombia (CO) with all current sequences (12/2018) available from GenBank database. Sequences were aligned using the program MAFFT-L-INS-i [1, See S1 Text.] and manual curated. Sequence labels consist of country code, accession number and genotype; BR Brazil, DE Germany, IL Israel, IT Italy, MT Malta, JP Japan. The TCG microsatellite triplet repeat and nucleotide polymorphisms served to discriminate between the different *Aelurostrongylus* genotypes are highlighted. Genotype A is found worldwide and is identical with *Ael*. *abstrusus*, whereas genotypes AB, B, and C were so far only described from Colombia.
(PDF)

S4 Fig Corresponding phylogenetic tree of *Aelurostrongylus* genotypes (Maximum-Likelihood PhyML). Phylogenetic analysis using phylogeny.fr web service [2, See S1 Text.], *Metastrongylus salmi* as outgroup, branches support values in percent with values < 50% not shown, scale-bar indicates the number of substitutions per site).
(PDF)

S1 Table Estimates of evolutionary divergence between *Aelurostrongylus* ITS2-Sequences with TGC-microsatellite. (PDF)

S2 Table Estimates of evolutionary divergence between *Aelurostrongyluso* ITS2-Sequences without the TGC-microsatellite. The number of base differences per site from between sequences are shown. This analysis involved 20 nucleotide sequences. All ambiguous positions were removed for each sequence pair (pairwise deletion option). There were a total of 449 positions in the final dataset. Evolutionary analyses were conducted in MEGA X [3. See S1Text.].
(PDF)

S1 Text Supporting information references. (DOCX)
