# Supplementary figures and images for: The invasive giant African snail Lissachatina fulica as natural intermediate host of Aelurostrongylus abstrusus, Angiostrongylus vasorum, Troglostrongylus brevior, and Crenosoma vulpis in Colombia
Source: PLoS Negl Trop Dis. 2019 Apr 19;13(4):e0007277. doi: 10.1371/journal.pntd.0007277 (PMC6493767; doi:10.1371/journal.pntd.0007277)

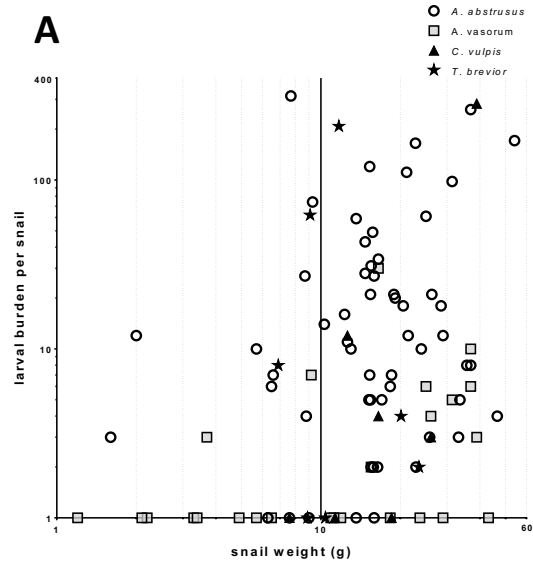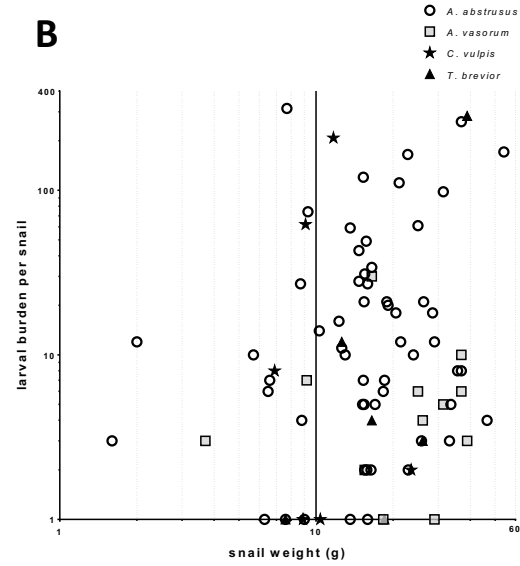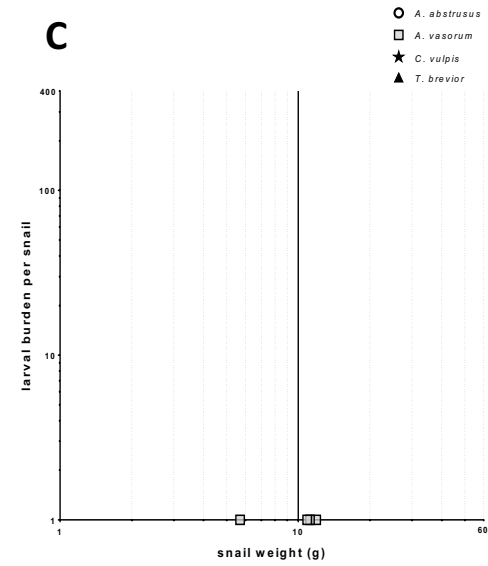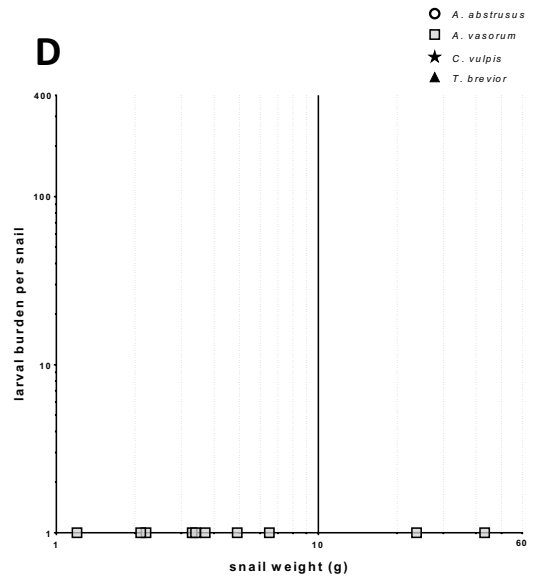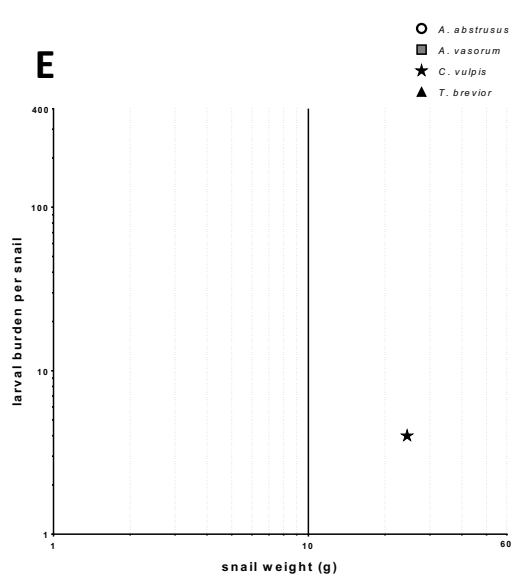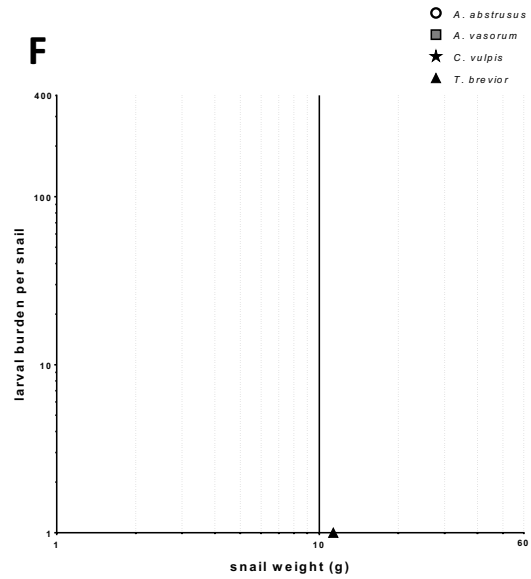

Supplement: S1 Fig — A) Larval burden per snail and snail weight of all 609 snails and those collected in B) Puerto Leguízamo (n = 107), C) Andes (n = 238), D) Tuluá (n = 64), E) Cañasgordas (n = 100) and F) and Ciudad Bolívar (n = 100). (PDF) [file pntd.0007277.s001.pdf]

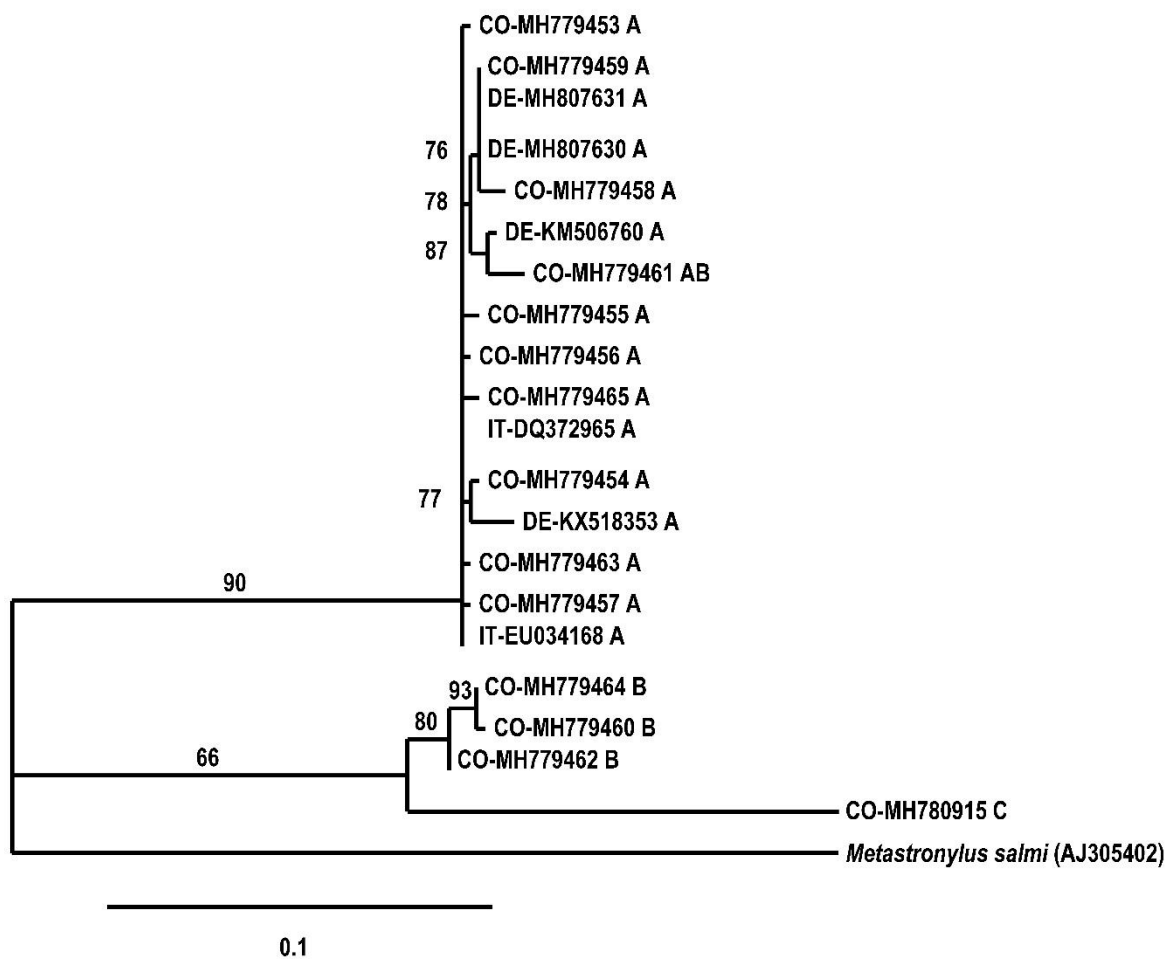

Supplement: S4 Fig — Phylogenetic analysis using phylogeny.fr web service [2, See S1 Text.], Metastrongylus salmi as outgroup, branches support values in percent with values < 50% not shown, scale-bar indicates the number of substitutions per site). (PDF) [file pntd.0007277.s004.pdf]
