# Supplementary material for: The invasive giant African snail Lissachatina fulica as natural intermediate host of Aelurostrongylus abstrusus, Angiostrongylus vasorum, Troglostrongylus brevior, and Crenosoma vulpis in Colombia
Source: PLoS Negl Trop Dis. 2019 Apr 19;13(4):e0007277. doi: 10.1371/journal.pntd.0007277 (PMC6493767; doi:10.1371/journal.pntd.0007277)
Supplement: S2 Fig — Alignment CO Aelurostrongylus genotypes A, AB, B and C. Alignment of ITS2 sequences from Ael. abstrusus isolates detected during the present study in Lisachatina fulica in Colombia (CO). Genotype A is found worldwide and is identical with European isolates of Ael. abstrusus, whereas genotypes AB, B, and unknown species C were so far only described from Colombia. (PDF) [file pntd.0007277.s002.pdf]

```
CO-MH779457 A     1 AGAAATTAGCAATACAGTTGTGTGTGATGTTGTACATTATGACTATATGCGACTGTTTGC
CO-MH779461 AB    1 AGAAATTAGCAATACAGTTGTGTGTGATGTTGTACATTATGACTATATGCGACTGTTTGC
CO-MH779464 B     1 AGAAATTAACAATACAGTTGTGTGTGATGTTGTACATTATGATTATATGTGACTGTTTGC
CO-MH780915 C     1                         TGATGTTGTGCATTATGGTTATATGTGACTGTTTGT

                    (TCG microsatellite)
                               1  2  3  4  5  6  7  8  9  10  *
CO-MH779457 A    61 CGAATGGTACTCGTCGTCGTCGTCGTCGTCGTCGTCGTCGTAGTCTTAGTCGTTATCGAC
CO-MH779461 AB   61 CGAATGGTACTCGTCGTCGTCGTCG---------------TAGTCTTAGTCGTTATCGAC
CO-MH779464 B    61 CGAATGGTACTCGTCGTCGTCG------------------TGGTCTTAGTCGTTATCGAC
CO-MH780915 C    37 AAAGTGGTACTCATCGTCGTCG---------------------TCTCAGTCGTTATCGAC

                                    *
CO-MH779457 A   121 TGACGATGTTGATGCGATCGATGATTCCCGTTTCAGTGAGGAATTAGATGAGAGCAACGT
CO-MH779461 AB  106 TGACGATGTTGATGCGATCGATGATTCCCGTTTCAGTGAGGAATTAGATGAGAGCAACGT
CO-MH779464 B   103 TGACGATGTTGATGCGCTCGATGATTCCCGTTTCAGTGAGGAATTAGATGAGAGCAACGT
CO-MH780915 C    76 TGACGG---TGATGCGATCGATGATTCCCGTTTTAGTGAGGGGTTAGATGAGAGCAACAT

                      ***         *             * *  *   *           *
CO-MH779457 A   181 GTAACAACGATATTGGTACTATGTTACATTGAGCGTAATGTGTATGTATGTGCATTTATG
CO-MH779461 AB  166 GTAACAACGATATTGGTACTATGTTACATTGAGCGTAATGTGTATGTATGTGCATTTATG
CO-MH779464 B   163 GT---AACGATATTAGTACTATGTTACACTAAGTGTATTGTGTATGTATATGCATTTATG
CO-MH780915 C   133 GT---AACGATATCGGTACTATGTTACACCA CTGTAATGTGTATGTAT-----TGTGTG

                                         *   *     ***
CO-MH779457 A   241 CTAGTGATATCATTACTATCATCGTTGATGTTGGTGGTTTTCAATGGGTATCGTTGAGAA
CO-MH779461 AB  226 CTAGTGATATCATTACTATCACCGTAGATGT---TGGTTTTCAATGGGTATCGTTGAGAA
CO-MH779464 B   220 CTAGTGATATCATTACTATCACCGTAGATGT---TGGTTTTCAATGGGTATCGTTGAGAA
CO-MH780915 C   185 CTAGTGATATCATTACTATCATCGCAGATGT---TGTTTTTCAATGGATATCGTTGAGAA

CO-MH779457 A   301 TCGTGAATTGGAGAACATCGTTGACGATGAATGGCAACGACACGTGGTTGGTTTTGAGTA
CO-MH779461 AB  283 TCGTGAATTGGAGAACATCGTTGACGATGAATGACAACGACATGTGGTTGGTTTTGAGTA
CO-MH779464 B   277 TCGTGAATTGGAGAACATCGTTGACGATGAATGACAACGACATGTGGTTGGTTTTGAGTA
CO-MH780915 C   242 TCGTGAATTGGAGAACATTGTTGACGATGAATTACAACGACATGTGGTTGGTTTCGAGTA

CO-MH779457 A   361 ATGATGATGACTAAATACACTAGGCATATTGCGAGTAGCACGTGAAGGAGTTCACATTGC
CO-MH779461 AB  343 ATGATGACGACTAAATACACTAGACATATTGCGAGT---ACGTGAAGGAGTTCACATTAC
CO-MH779464 B   337 ATGATGACGACTAAATACACTAGACATATTGCGAGTAGCACGTGAAGGAGTTCACATTGC
CO-MH780915 C   302 ATGATGACGACTAAATACACTAGGCATACTGTAAGTAGCACGTGCAGCAGTTCACACTAC

CO-MH779457 A   421 ATGTAATTCTTGCAAATGCAACCTGAACTCGGATGTGATTACCCGCTGAACT
CO-MH779461 AB  400 ATGTAATTCTTGCAAATGCTACCTGAACTCGGATGTGATTACCCGCTGAACT
CO-MH779464 B   397 ATGTAATTCTTGCAAATGCAACCTGAACTCGGATGTGATTACCCGCTGAACT
CO-MH780915 C   362 GTGTAGTTCTTGCAGATA AACCTAAACTAGAATGTGATTACCCGCTGAACT
```

Nucleotide polymorphisms conserved in haplotype B sequences are marked by an asterisk (*)
