# Supplementary material for: The invasive giant African snail Lissachatina fulica as natural intermediate host of Aelurostrongylus abstrusus, Angiostrongylus vasorum, Troglostrongylus brevior, and Crenosoma vulpis in Colombia
Source: PLoS Negl Trop Dis. 2019 Apr 19;13(4):e0007277. doi: 10.1371/journal.pntd.0007277 (PMC6493767; doi:10.1371/journal.pntd.0007277)
Supplement: S3 Fig — Alignment of ITS2 sequences from Ael. abstrusus isolates detected in Colombia (CO) with all current sequences (12/2018) available from GenBank database. Sequences were aligned using the program MAFFT-L-INS-i [1, See S1 Text.] and manual curated. Sequence labels consist of country code, accession number and genotype; BR Brazil, DE Germany, IL Israel, IT Italy, MT Malta, JP Japan. The TCG microsatellite triplet repeat and nucleotide polymorphisms served to discriminate between the different Aelurostrongylus genotypes are highlighted. Genotype A is found worldwide and is identical with Ael. abstrusus, whereas genotypes AB, B, and C were so far only described from Colombia. (PDF) [file pntd.0007277.s003.pdf]

```
IT-DQ372965 A     1 AGAAATTAGCAATACAGTTGTGTGTGATGTTGTACATTATGACTATATGCGACTGTTTGC
CO-MH779457 A     1 AGAAATTAGCAATACAGTTGTGTGTGATGTTGTACATTATGACTATATGCGACTGTTTGC
IT-EU034168 A     1 AGAAATTAGCAATACAGTTGTGTGTGATGTTGTACATTATGACTATATGCGACTGTTTGC
CO-MH779456 A     1 AGAAATTAGCAATACAGTTGTGTGTGATGTTGTACATTATGACTATATGCGACTGTTTGC
CO-MH779453 A     1 AGAAATTAGCAATACAGTTGTGTGTGATGTTGTACATTATGACTATATGCGACTGTTTGC
CO-MH779463 A     1 AGAAATTAGCAATACAGTTGTGTGTGATGTTGTACATTATGACTATATGCGACTGTTTGC
CO-MH779455 A     1 AGAAATTAGCAATACAGTTGTGTGTGATGTTGTACATTATGACTATATGCGACTGTTTGC
CO-MH779465 A     1 AGAAATTAGCAATACAGTTGTGTGTGATGTTGTACATTATGACTATATGCGACTGTTTGC
DE-KM506760 A     1 AGAAATTAGCAATACAGTTGTGTGTGATGTTGTACATTATGACTATATGCGACTGTTTGC
CO-MH779454 A     1 AGAAATTAGCAATACAGTTGTGTGTGATGTTGTACATTATGACTATATGCGACTGTTTGC
DE-MH807631 A     1 AGAAATTAGCAATACAGTTGTGTGTGATGTTGTACATTATGACTATATGCGACTGTTTGC
CO-MH779459 A     1 AGAAATTAGCAATACAGTTGTGTGTGATGTTGTACATTATGACTATATGCGACTGTTTGC
DE-MH807630 A     1 AGAAATTAGCAATACAGTTGTGTGTGATGTTGTACATTATGACTATATGCGACTGTTTGC
DE-KX518353 A     1 AGAAATTAGCAATACAGTTGTGTGTGATGTTGTACATTATGACTATATGCGACTGTTTGC
CO-MH779458 A     1 AGAAATTAGC-ATACAGTTGTGTGTGATGTTGTACATTATGACTATATGCGACTGTTTGC
CO-MH779461 AB    1 AGAAATTAGCAATACAGTTGTGTGTGATGTTGTACATTATGACTATATGCGACTGTTTGC
CO-MH779462 B     1 AGAAATTAGCAATACAGTTGTGTGTGATGTTGTACATTATGATTATATGTGACTGTTTGC
CO-MH779464 B     1 AGAAATTAACAATACAGTTGTGTGTGATGTTGTACATTATGATTATATGTGACTGTTTGC
CO-MH779460 B     1 AGAAATTAACAATACAGTTGTGTGTGATGTTGTACATTATGATTATATGTGACTGTTTGC
CO-MH780915 C     1                         TGATGTTGTGCATTATGGTTATATGTGACTGTTTGT
```

(TCG-microsatellite)

```
                               1  2  3  4  5  6  7  8  9  10
IT-DQ372965 A    61 CGAATGGTACTCGTCGTCGTCGTCGTCGTCGTCGTCGTCGTAGTCTTAGTCGTTATCGAC
IT-JX948745 A     1       GTACTAGTCGTCGTCGTCGTCGTCGTCGTCGTCGTAGTCTTAGTCGTTATCGAC
CO-MH779457 A    61 CGAATGGTACTCGTCGTCGTCGTCGTCGTCGTCGTCGTCGTAGTCTTAGTCGTTATCGAC
IT-EU034168 A    61 CGAATGGTACTCGTCGTCGTCGTCGTCGTCGTCGTCGTCGTAGTCTTAGTCGTTATCGAC
CO-MH779456 A    61 CGAATGGTACTCGTCGTCGTCGTCGTCGTCGTCG------TAGTCTTAGTCGTTATCGAC
CO-MH779453 A    61 CGAATGGTACTCGTCGTCGTCGTCGTCGTCGTCG------TAGTCTTAGTCGTTATCGAC
CO-MH779463 A    61 CGAATGGTACTCGTCGTCGTCGTCGTCGTCGTCGTCGTCGTAGTCTTAGTCGTTATCGAC
CO-MH779455 A    61 CGAATGGTACTCGTCGTCGTCGTCGTCGTCGTCGTCG---TAGTCTTAGTCGTTATCGAC
CO-MH779465 A    61 CGAATGGTACTCGTCGTCGTCGTCGTCGTCG---------TAGTCTTAGTCGTTATCGAC
DE-KM506760 A    61 CGAATGGTACTCGTCGTCGTCGTCGTCGTCGTCGTCGTCGTAGTCTTAGTCGTTATCGAC
CO-MH779454 A    61 CGAATGGTACTCGTCGTCGTCGTCGTCGTCGTCG------TAGTCTTAGTCGTTATCGAC
DE-MH807631 A    61 CGAATGGTACTCGTCGTCGTCGTCGTCGTCGTCG------TAGTCTTAGTCGTTATCGAC
CO-MH779459 A    61 CGAATGGTACTCGTCGTCGTCGTCGTCGTCGTCG------TAGTCTTAGTCGTTATCGAC
DE-MH807630 A    61 CGAATGGTACTCGTCGTCGTCGTCGTCGTCG---------TAGTCTTAGTCGTTATCGAC
DE-KX518353 A    61 CGAATGGTACTCGTCGTCGTCGTCGTCGTCGTCGTCG---TAGTGTTAGTCGTTATCGAC
BR-DQ029000 A     1       CTAGTGGTCGTCGTCGTCGTCGTCGTCGTCG---TAGTCTTAGTCGTTATCGAC
CO-MH779458 A    60 CGAATGGTAC--GTCGTCGTCGTCGTCGTCGTCG------TAGTCTTAGTCGTTATCGAC
CO-MH779461 AB   61 CGAATGGTACTCGTCGTCGTCGTCG---------------TAGTCTTAGTCGTTATCGAC
CO-MH779462 B    61 CGAATGGTACTCGTCGTCGTCG------------------TGGTCTTAGTCGTTATCGAC
CO-MH779464 B    61 CGAATGGTACTCGTCGTCGTCG------------------TGGTCTTAGTCGTTATCGAC
CO-MH779460 B    61 CGAATGGTACTCGTCGTCGTCG------------------TGGTCTTAGTCGTTATCGAC
CO-MH780915 C    37 AAAGTGGTACTCATCGTCGTCG---------------------TCTCAGTCGTTATCGAC
```

```
IT-DQ372965 A   121 TGACGATGTTGAT-GCGATCGATGATTCCCGTTTCAGTGAGGAATTAGATGAGAGCAACG
IT-KM009116 A     1                                    AGTGAGGAATTAGATGAGAGCAACG
IT-JX948745 A    55 TGACGATGTTGAT-GCGATCGATGATTCCCGTTTCAGTGAGGAATTAGATGAGAGCAACG
CO-MH779457 A   121 TGACGATGTTGAT-GCGATCGATGATTCCCGTTTCAGTGAGGAATTAGATGAGAGCAACG
IT-EU034168 A   121 TGACGATGTTGAT-GCGATCGATGATTCCCGTTTCAGTGAGGAATTAGATGAGAGCAACG
CO-MH779456 A   115 TGACGATGTTGAT-GCGATCGATGATTCCCGTTTCAGTGAGGAATTAGATGAGAGCAACG
CO-MH779453 A   115 TGACGATGTTGAT-GCGATCGATGATTCCCGTTTCAGTGAGGAATTAGATGAGAGCAACG
CO-MH779463 A   121 TGACGATGTTGAT-GCGATCGATGATTCCCGTTTCAGTGAGGAATTAGATGAGAGCAACG
CO-MH779455 A   118 TGACGATGTTGAT-GCGATCGATGATTCCCGTTTCAGTGAGGAATTAGATGAGAGCAACG
CO-MH779465 A   112 TGACGATGTTGAT-GCGATCGATGATTCCCGTTTCAGTGAGGAATTAGATGAGAGCAACA
DE-KM506760 A   121 TGACGATGTTGAT-GCGATCGATGGTTCCCGTTTCAGTGAGGAATTAGATGAGAGCAACG
CO-MH779454 A   115 TGACGATGTTGAT-GCGATCGATGATTCCCGTTTCAGTGAGGAATTAGAGGAGAGCAACG
DE-MH807631 A   115 TGACGATGTTGATGGCGATCGATGATTCCCGTTTCAGTGAGGAATTAGATGAGAGCAACG
CO-MH779459 A   115 TGACGATGTTGATGGCGATCGATGATTCCCGTTTCAGTGAGGAATTAGATGAGAGCAACG
DE-MH807630 A   112 TGACGATGTTGATGGCGATCGATGATTCCCGTTTCAGTGAGGAATTAGATGAGAGCAACG
DE-KX518353 A   118 TGACGATGTTGAT-GCGATCGATGATTCCCGTTTCAGTGAGGAATTAGATGAGGGCAACG
BR-DQ029000 A    52 TGACGATGTTGAT-GCGATCGATGATTCCCGTTTCAGTGAGGAATTAGATGAGAGCAACG
CO-MH779458 A   112 TGACGATGTTGAT-GCGATCGATGATTCCCGTTCCAGTGAGGAATTAGATGAGAGCAACG
CO-MH779461 AB  106 TGACGATGTTGAT-GCGATCGATGATTCCCGTTTCAGTGAGGAATTAGATGAGAGCAACG
CO-MH779462 B   103 TGACGATGTTGAT-GCGCTCGATGATTCCCGTTTCAGTGAGGAATTAGATGAGAGCAACG
CO-MH779464 B   103 TGACGATGTTGAT-GCGCTCGATGATTCCCGTTTCAGTGAGGAATTAGATGAGAGCAACG
CO-MH779460 B   103 TGACGATGTTGAT-GCGCTCGATGATTCCCGTTTCAGTGAGGAATTAGATGAGAGCAACG
CO-MH780915 C    76 TGACGG---TGAT-GCGATCGATGATTCCCGTTTTAGTGAGGGGTTAGATGAGAGCAACA
```

```
IT-DQ372965 A   180 TGTAACAACGATATTGGTACTATGTTACATTGAGCGTAATGTGTATGTATGTGCATTTAT
IT-KM009116 A    26 TGTAACAACGATATTGGTACTATGTTACATTGAGCGTAATGTGTATGTATGTGCATTTAT
IT-JX948745 A   114 TGTAACAACGATATTGGTACTATGTTACATTGAGCGTAATGTGTATGTATGTGCATTTAT
CO-MH779457 A   180 TGTAACAACGATATTGGTACTATGTTACATTGAGCGTAATGTGTATGTATGTGCATTTAT
IT-EU034168 A   180 TGTAACAACGATATTGGTACTATGTTACATTGAGCGTAATGTGTATGTATGTGCATTTAT
CO-MH779456 A   174 TGTAACAACGATATTGGTACTATGTTACATTGGGCGTAATGTGTATGTATGTGCATTTAT
CO-MH779453 A   174 TGTAACAACGATATTGGTACTATGTTACATTGAGCGTAATGTGTATGTATGTGCATTTAT
CO-MH779463 A   180 TGTAACAACGATATTGGTACTATGTTACATTGAGCGTAATGTGTATGTATGTGCATTTAT
CO-MH779455 A   177 TGTAATAACGATATTGGTACTATGTTACATTGAGCGTAATGTGTATGTATGTGCATTTAT
CO-MH779465 A   171 TGTAACAACGATATTGGTACTATGTTACATTGAGCGTAATGTGTATGTATGTGCATTTAT
DE-KM506760 A   180 TGTAACAACGATATTGGTACTATGTTACATTGAGCGTAATGTGTATGTATGTGCATTTAT
CO-MH779454 A   174 TGTAACAACGATATTGGTACTATGTTACATTGAGCGTAATGTGTATGTATGTGCATTTAT
DE-MH807631 A   175 TGTAACAACGATATTGGTACTATGTTACATTGAGCGTAATGTGTATGTATGTGCATTTAT
CO-MH779459 A   175 TGTAACAACGATATTGGTACTATGTTACATTGAGCGTAATGTGTATGTATGTGCATTTAT
DE-MH807630 A   172 TGTAACAACGATATTGGTACTATGTTACATTGAGCGTAATGTGTATGTATGTGCATTTAT
DE-KX518353 A   177 TGTAACAACGATATTGGTACTATGTTACATTGAGCGTAATGTGTATGTGTGTGCATTTAT
BR-DQ029000 A   111 TGTAACAACGATATTGGTACTATGTTACATTGAGCGTAATGTGTATGTATGTGCATTTAT
CO-MH779458 A   171 TGTAACAAAGATATTGGTACTATGTTACATTGAGCGTAATGTGTATGTATGTGCATTTAT
IL-MH593881 A     1    AACAACGATATTGGTACTATGTTACATTGAGCGTAATGTGTATGTATGTGCATTTAT
CO-MH779461 AB  165 TGTAACAACGATATTGGTACTATGTTACATTGAGCGTAATGTGTATGTATGTGCATTTAT
CO-MH779462 B   162 TGT---AACGATATTAGTACTATGTTACACTAAGTGTATTGTGTATGTATATGCATTTAT
CO-MH779464 B   162 TGT---AACGATATTAGTACTATGTTACACTAAGTGTATTGTGTATGTATATGCATTTAT
CO-MH779460 B   162 TGT---AACGATATTAGTACTATGTTACACTAAGTGTATTGTGTATGTATATGCATTTAT
CO-MH780915 C   132 TGT---AACGATATCGGTACTATGTTACACCAACTGTAATGTGTATGTAT-----TGTGT

IT-DQ372965 A   240 GCTAGTGATATCATTACTATCATCGTTGATGTTGGTGGTTTTCAATGGGTATCGTTGAGA
IT-KM009116 A    86 GCTAGTGATATCATTACTATCATCGTTGATGTTGGTGGTTTTCAATGGGTATCGTTGAGA
IT-JX948745 A   174 GCTAGTGATATCATTACTATCATCGTTGATGTTGGTGGTTTTCAATGGGTATCGTTGAGA
CO-MH779457 A   240 GCTAGTGATATCATTACTATCATCGTTGATGTTGGTGGTTTTCAATGGGTATCGTTGAGA
IT-EU034168 A   240 GCTAGTGATATCATTACTATCATCGTTGATGTTGGTGGTTTTCAATGGGTATCGTTGAGA
CO-MH779456 A   234 GCTAGTGATATCATTACTATCATCGTTGATGTTGGTGGTTTTCAATGGGTATCGTTGAGA
MT-MF593917 A     1              ATTACTATCATCGTTGATGTTGGTGGTTTTCAATGGGTATCGTTGAGA
IT-KF751655 A     1              ATTACTATCATCGTTGATGTTGGTGGTTTTCAATGGGTATCGTTGAGA
CO-MH779453 A   234 GCTAGTGATATCATTACTATCATCGTTGATGTTGATGGTTTTCAATGGGTATCGTTGAGA
CO-MH779463 A   240 GCTAGTGATATCATTACTATCATCGTTGATGTTGGTGGTTTTCAATGGGTATCGTTGAGA
CO-MH779455 A   237 GCTAGTGATATCATTACTGTCATCGTTGATGTTGGTGGTTTTCAATGGGTATCGTTGAGA
CO-MH779465 A   231 GCTAGTGATATCATTACTATCATCGTTGACGTTGGTGGTTTTCAATGGGTATCGTTGAGA
DE-KM506760 A   240 GCTAGTGATATCATTACTATCATCGTTGATGTTGGTGGTTTTCAATGGGTATCGTTGAGA
CO-MH779454 A   234 GCTAGTGATATCATTACTATCATCGTTGATGTTGGTGGTTTTCAATGGGTATCGTTGAGA
DE-MH807631 A   235 GCTAGTGATATCATTACTATCATCGTTGATGTTGGTGGTTTTCAATGGGTATCGTTGAGA
CO-MH779459 A   235 GCTAGTGATATCATTACTATCATCGTTGATGTTGGTGGTTTTCAATGGGTATCGTTGAGA
DE-MH807630 A   232 GCTAGTGATATCATTACTATCATCGTTGATGTTGGTGGTTTTCAATGGGTATCGTTGAGA
DE-KX518353 A   237 GCTAGTGATATCATTACTATCATCGTTGATGTTGGCGGTTTTCAATGGGTATCGTTGAGA
BR-DQ029000 A   171 GCTAGTGATATCATCACTATCATCGTTGAT
CO-MH779458 A   231 GCTAGTGATATCATTACTATCATCGTTGATGTTGGTGGTTTTCAATGGGTATCGTTGAGA
IL-MH593881 A    58 GCTAGTGATATCATTACTATCATCGTTGATGTTGGTGGTTTTCAATGGGTATCGTTGAGA
JP-KY774310 A     1              ATTACTATCATCGTTGATGTTGGTGGTTGTCAATGGGTATCGTTGAGA
CO-MH779461 AB  225 GCTAGTGATATCATTACTATCACCGTAGATGT---TGGTTTTCAATGGGTATCGTTGAGA
CO-MH779462 B   219 GCTAGTGATATCATTACTATCACCGTAGATGT---TGGTTTTCAATGGGTATCGTTGAGA
CO-MH779464 B   219 GCTAGTGATATCATTACTATCACCGTAGATGT---TGGTTTTCAATGGGTATCGTTGAGA
CO-MH779460 B   219 GCTAGTGATATCATTACTATCACCGTAGATGT---TGGTTTTCAATGGGTATCGTTGAGA
CO-MH780915 C   184 GCTAGTGATATCATTACTATCATCGCAGATGT---TGTTTTTCAATGGATATCGTTGAGA

IT-DQ372965 A   300 ATCGTGAATTGGAGAACATCGTTGACGATGAATGACAACGACACGTGGTTGGTTTTGAGT
IT-KM009116 A   146 ATCGTGAATTGGAGAACATCGTTGACGATGAATGACAACGACACGTGGTTGGTTTTGAGT
IT-JX948745 A   234 ATCGTGAATTGGAGAACATCGTTGACGATGAATGACAACGACACGTGGTTGGTTTTGAGT
CO-MH779457 A   300 ATCGTGAATTGGAGAACATCGTTGACGATGAATGGCAACGACACGTGGTTGGTTTTGAGT
IT-EU034168 A   300 ATYGTGAATTGGAGAACATCGTTGACGATGAATGACAAYGACACGTGGTTGGTTTTGAGT
CO-MH779456 A   294 ATCGTGAATTGGAGAACATCGTTGACGATGAATGACAACGACACGTGGTTGGTTTTGAGT
MT-MF593917 A    49 ATCGTGAATTGGAGAACATCGTTGACGATGAATGACAACGACACGTGGTTGGTTTTGAGT
IT-KF751655 A    49 ATCGTGAATTGGAGAACATCGTTGACGATGAATGACAACGACACGTGGTTGGTTTTGAGT
CO-MH779453 A   294 ATCGTGAATTGGAGAACATCGTTGACGATGAATGACAACGACACGTGGTTGGTTTTGAGT
CO-MH779463 A   300 ATCGTGAATTGGAGAACATCGTTGACGATGAATGACAACGACACGTGGTTGGTTTTGAGT
CO-MH779455 A   297 ATCGTGAATTGGAGAACATCGTTGACGATGAATGACAACGACACGTGGTTGGTTTTGAGT
CO-MH779465 A   291 ATCGTGAATTGGAGAACATCGTTGACGATGAATGACAACGACACGTGGTTGGTTTTGAGT
DE-KM506760 A   300 ATCGTGAATTGGAGAACATCGTTGACGATGAATGACAACGACACGTGGTTGGTTTTGAGT
CO-MH779454 A   294 ATCGTGAATTGCAGAACATCGTTGACGATGAATGACAACGACACGTGGTTGGTTTTGAGT
DE-MH807631 A   295 ATCGTGAATTGGAGAACATCGTTGACGATGAATGACAACGACACGTGGTTGGTTTTGAGT
CO-MH779459 A   295 ATCGTGAATTGGAGAACATCGTTGACGATGAATGACAACGACACGTGGTTGGTTTTGAGT
DE-MH807630 A   292 ATCGTGAATTGGAGAACATCGTTGACGATGAATGACAACGACACGTGGTTGGTTTTGAGT
DE-KX518353 A   297 ATCGTGAATTGCAGAACATCGTTGACGATGAATGACAACGACACGCGGTTGGTTTTGAGT
CO-MH779458 A   291 ATCGTGAATTGGAGAACATCGTTGACGATGAATGACAACGACACGTGGTTGGTTTTGAGT
IL-MH593881 A   118 ATCGTGAATTGGAGAACATCGTTGACGATGAATGACAACGACACGTGGTTGGTTTTGAGT
JP-KY774310 A    49 ATCGTGAATTGGAGAACATCGTTGACGATGAATGACAACGACACGTGGTTGGCTTTGACT
CO-MH779461 AB  282 ATCGTGAATTGGAGAACATCGTTGACGATGAATGACAACGACATGTGGTTGGTTTTGAGT
```

```
CO-MH779462 B  276 ATCGTGAATTGGAGAACATCGTTGACGATGAATGACAACGACACGTGGTTGGTTTTGAGT
CO-MH779464 B  276 ATCGTGAATTGGAGAACATCGTTGACGATGAATGACAACGACATGTGGTTGGTTTTGAGT
CO-MH779460 B  276 ATCGTGAATTGGAGAACATCGTTGACGATGAATGACAACGACATGTGGTTGGTTTTGAGT
CO-MH780915 C  241 ATCGTGAATTGGAGAACATTGTTGACGATGAATTACAACGACATGTGGTTGGTTTCGAGT

IT-DQ372965 A  360 AATGATGATGACTAAATACACTAGGCATATTGCGAGTAGCACGTGAAGG-AGTTCACATT
IT-KM009116 A  206 AATGATGATGACTAAATACACTAGGCATATTGCGAGTAGCACGTGAAGG-AGTTCACATT
IT-JX948745 A  294 AATGATGATGACTAAATACACTAGGCATATTGCGAGTAGCACGTGAAGG-AGTTCACATT
CO-MH779457 A  360 AATGATGATGACTAAATACACTAGGCATATTGCGAGTAGCACGTGAAGG-AGTTCACATT
IT-EU034168 A  360 AATGATGATGACTAAATACACTAGGCATATTGCGAGTAGCACGTGAAGG-AGTTCACATT
CO-MH779456 A  354 AATGATGATGACTAAATACACTAGGCATATTGCGAGTAGCACGTGAAGG-AGTTCACATT
MT-MF593917 A  109 AATGATGATGACTAAATACACTAGGCATATTGCGAGTAGCACGTGAAGG-AGTTCACATT
IT-KF751655 A  109 AATGATGATGACTAAATACACTAGGCATATTGCGAGTAGCACGTGAAGG-AGTTCACATT
CO-MH779453 A  354 AATGATGATGACTAAATACACTAGGCATATTGCGAGTAGCACGTGAAGG-AGTTCACATT
CO-MH779463 A  360 AATGATGATGACTAAATACACTAGGCATATTGCGAGTAGCACGTGAAGG-AGTTCACATT
CO-MH779455 A  357 AATGATGATGACTAAATACACTAGGCATATTGCGAGTAGCACGTGAAGG-AGTTCACATT
CO-MH779465 A  351 AATGATGATGACTAAATACACTAGGCATATTGCGAGTAGCACGTGAAGG-AGTTCACATT
DE-KM506760 A  360 AATGATGACGACTAAATACACTAGACATATTGCGAGTAGCACGTGAAGG-AGTTCACATT
CO-MH779454 A  354 AATGATGATGACTAAATACACTAGGCATATTGCGAGTAGCACGTGAAGG-AGTTCACATT
DE-MH807631 A  355 AATGATGACGACTAAATACACTAGGCATATTGCGAGTAGCACGTGAAGG-CGTTCACATT
CO-MH779459 A  355 AATGATGACGACTAAATACACTAGGCATATTGCGAGTAGCACGTGAAGG-CGTTCACATT
DE-MH807630 A  352 AATGATGACGACTAAATACACTAGGCATATTGCGAGTAGCACGTGAAGG-CGTTCACATT
DE-KX518353 A  357 AATGATGATGACTAAATACACTAGGCATATTGCGAGTAGCACGTGAAGG-AGTTCACATT
CO-MH779458 A  351 AATGATGACGGCTAAATACACTAGGCATATTGCGAGTAGCACGTGAAGG-CGTTCACATT
IL-MH593881 A  178 AATGATGATGACTAAATACACTAGGCATATTGCGAGTAGCACGTGAAGGCAGTTCACATT
JP-KY774310 A  109 AATGATGATGACTAAATACACTAGGCATATTGCGAGTAGCTCGTGAAGG-AGTTCACATT
CO-MH779461 AB 342 AATGATGACGACTAAATACACTAGACATATTGCGAGT---ACGTGAAGG-AGTTCACATT
CO-MH779462 B  336 AATGATGATGACTAAATACACTAGGCATATTGCGAGTAGCACGTGAAGG-AGTTCACATT
CO-MH779464 B  336 AATGATGACGACTAAATACACTAGACATATTGCGAGTAGCACGTGAAGG-AGTTCACATT
CO-MH779460 B  336 AATGATGACGACTAAATACACTAGACATATTGCGAGT---ACGTGAAGG-AGTTCACATT
CO-MH780915 C  301 AATGATGACGACTAAATACACTAGGCATACTGTAAGTAGCACGTGCAGC-AGTTCACACT

IT-DQ372965 A  419 GCATGTAATTCTTGCAAATGCAACCTGAACTCGGATGTGATTACCCGCTGAACT
IT-KM009116 A  265 GCATGTAATTCTTGCAAATGCAACCTGAACTCGGATGTGA
IT-JX948745 A  353 GCATGTAATTCTTGCAAATGCAACCTGAACTCGGATGTGATACCC
CO-MH779457 A  419 GCATGTAATTCTTGCAAATGCAACCTGAACTCGGATGTGATTACCCGCTGAACT
IT-EU034168 A  419 GCATGTAATTCTTGCAAATGCAACCTGAACTCGGATGTGATTACCCGCTGAACT
CO-MH779456 A  413 GCATGTAATTCTTGCAAATGCAACCTGAACTCGGATGTGATTACCCGCTGAACT
MT-MF593917 A  168 GCATGTAATTCTTGCAAATGCAACCTGAACTCGGATGTGATTACCCGCTGAACT
IT-KF751655 A  168 GCATGTAATTCTTGCAAATGCAACCTGAACTCGGATGTGATTACCCGCTGAACT
CO-MH779453 A  413 GCATGTAATTCTTGCAAATGCAACCTGAACTCGGATGTGATTACCCGCTGAACT
CO-MH779463 A  419 GCATGTAATTCTTGCAAATGCAACCTGAACTCGGTTGTGATTACCCGCTGAACT
CO-MH779455 A  416 GCATGTAATTCTTGCAAATGCAACCTGAACTCGGATGTGATTACCCGCTGAACT
CO-MH779465 A  410 GCATGTAATTCTTGCAAATGCAACCTGAACTCGGATGTGATTACCCGCTGAACT
DE-KM506760 A  419 ACATGTAATTCTTGCAAATGCAACCTGAACTCGGATGTGATTACCCGCTGAACT
CO-MH779454 A  413 GCATGTAATTCTTGCAAATGCAACCTGAACTCGGATGTGATTACCCGCTGAACT
DE-MH807631 A  414 GCATGTAATTCTTGCAAATGCAACCTGAACTCGGATGTGATTACCCGCTGAACT
CO-MH779459 A  414 GCATGTAATTCTTGCAAATGCAACCTGAACTCGGATGTGATTACCCGCTGAACT
DE-MH807630 A  411 GCATGTAATTCTTGCAAATGCAACCTGAACTCGGATGTGATTACCCGCTGAACT
DE-KX518353 A  416 GCATGTAATTCTTGCAAATGCAACCTGAACTCGGATGTGATTACCCGCTGAACT
CO-MH779458 A  410 GCATGTAATTCTTGCAAATGCAACCTGAACTCGGATGTGATTACCCGCTGAACT
IL-MH593881 A  238 GCATGTAATTCTTGCAAATGCAACCTGAACTCGGATGTGATTACCCGCTGAACT
JP-KY774310 A  168 GCATGTAATTCTTGCAAATGCAACCTGAACTCGGATGTGATTACCCGCTGAAC-
CO-MH779461 AB 398 ACATGTAATTCTTGCAAATGCTACCTGAACTCGGATGTGATTACCCGCTGAACT
CO-MH779462 B  395 GCATGTAATTCTTGCAAATGCAACCTGAACTCGGATGTGATTACCCGCTGAACT
CO-MH779464 B  395 GCATGTAATTCTTGCAAATGCAACCTGAACTCGGATGTGATTACCCGCTGAACT
CO-MH779460 B  392 ACATGTAATTCTTGCAAATGCAACCTGAACTCGGATGTGATTACCCGCTGAACT
CO-MH780915 C  360 ACGTGTAGTTCTTGCAGATACAACCTAAACTAGAATGTGATTACCCGCTGAACT
```
