# Supplementary material for: The invasive giant African snail Lissachatina fulica as natural intermediate host of Aelurostrongylus abstrusus, Angiostrongylus vasorum, Troglostrongylus brevior, and Crenosoma vulpis in Colombia
Source: PLoS Negl Trop Dis. 2019 Apr 19;13(4):e0007277. doi: 10.1371/journal.pntd.0007277 (PMC6493767; doi:10.1371/journal.pntd.0007277)
Supplement: S1 Table — (PDF) [file pntd.0007277.s005.pdf]

	Country-Acc. No.	Geno-type	1	2	3	4	5	6	7	8	9	10	11	12	13	14	15	16	17	18	19
1	IT-DQ372965	A																			
2	CO-MH779457	A	0,002																		
3	IT-EU034168	A	0,000	0,002																	
4	CO-MH779463	A	0,002	0,004	0,002																
5	DE-KM506760	A	0,009	0,011	0,009	0,011															
6	CO-MH779455	A	0,004	0,007	0,005	0,007	0,013														
7	DE-KX518353	A	0,013	0,016	0,014	0,016	0,022	0,018													
8	CO-MH779456	A	0,002	0,005	0,002	0,005	0,011	0,007	0,016												
9	CO-MH779453	A	0,002	0,005	0,002	0,005	0,011	0,007	0,016	0,005											
10	CO-MH779454	A	0,005	0,007	0,005	0,007	0,014	0,009	0,014	0,007	0,007										
11	CO-MH779465	A	0,005	0,007	0,005	0,007	0,014	0,009	0,018	0,007	0,007	0,009									
12	DE-MH807631	A	0,005	0,007	0,005	0,007	0,009	0,009	0,018	0,007	0,007	0,009	0,009								
13	CO-MH779459	A	0,005	0,007	0,005	0,007	0,009	0,009	0,018	0,007	0,007	0,009	0,009	0,000							
14	DE-MH807630	A	0,005	0,007	0,005	0,007	0,009	0,009	0,018	0,007	0,007	0,009	0,009	0,000	0,000						
15	CO-MH779458	A	0,011	0,014	0,011	0,014	0,016	0,016	0,025	0,014	0,014	0,016	0,016	0,007	0,007	0,007					
16	CO-MH779461	AB	0,016	0,019	0,016	0,019	0,012	0,021	0,030	0,019	0,016	0,021	0,021	0,016	0,016	0,016	0,023				
17	CO-MH779462	B	0,028	0,031	0,028	0,031	0,038	0,031	0,042	0,031	0,028	0,033	0,033	0,033	0,033	0,033	0,040	0,036			
18	CO-MH779464	B	0,035	0,038	0,036	0,038	0,035	0,038	0,050	0,038	0,035	0,040	0,040	0,035	0,035	0,035	0,042	0,029	0,007		
19	CO-MH779460	B	0,038	0,040	0,038	0,040	0,033	0,040	0,052	0,040	0,038	0,043	0,043	0,038	0,038	0,038	0,045	0,026	0,010	0,002	
20	CO-MH780915	C	0,111	0,114	0,112	0,114	0,111	0,114	0,126	0,114	0,111	0,116	0,111	0,111	0,111	0,111	0,119	0,110	0,107	0,104	0,102
