# Supplementary material for: The invasive giant African snail Lissachatina fulica as natural intermediate host of Aelurostrongylus abstrusus, Angiostrongylus vasorum, Troglostrongylus brevior, and Crenosoma vulpis in Colombia
Source: PLoS Negl Trop Dis. 2019 Apr 19;13(4):e0007277. doi: 10.1371/journal.pntd.0007277 (PMC6493767; doi:10.1371/journal.pntd.0007277)
Supplement: S2 Table — The number of base differences per site from between sequences are shown. This analysis involved 20 nucleotide sequences. All ambiguous positions were removed for each sequence pair (pairwise deletion option). There were a total of 449 positions in the final dataset. Evolutionary analyses were conducted in MEGA X [3. See S1Text.]. (PDF) [file pntd.0007277.s006.pdf]

	Country-Acc. No.	Geno-type	1	2	3	4	5	6	7	8	9	10	11	12	13	14	15	16	17	18	19
1	IT-DQ372965	A																			
2	CO-MH779457	A	0,003																		
3	IT-EU034168	A	0,000	0,003																	
4	CO-MH779456	A	0,003	0,005	0,003																
5	CO-MH779453	A	0,003	0,005	0,003	0,005															
6	CO-MH779463	A	0,003	0,005	0,003	0,005	0,005														
7	CO-MH779455	A	0,005	0,008	0,005	0,008	0,008	0,008													
8	CO-MH779465	A	0,005	0,008	0,005	0,008	0,008	0,008	0,010												
9	DE-KM506760	A	0,010	0,013	0,010	0,013	0,013	0,013	0,015	0,015											
10	CO-MH779454	A	0,005	0,008	0,005	0,008	0,008	0,008	0,010	0,010	0,015										
11	DE-KX518353	A	0,013	0,015	0,013	0,015	0,015	0,015	0,018	0,018	0,023	0,013									
12	DE-MH807631	A	0,005	0,008	0,005	0,008	0,008	0,008	0,010	0,010	0,010	0,010	0,018								
13	CO-MH779459	A	0,005	0,008	0,005	0,008	0,008	0,008	0,010	0,010	0,010	0,010	0,018	0,000							
14	DE-MH807630	A	0,005	0,008	0,005	0,008	0,008	0,008	0,010	0,010	0,010	0,010	0,018	0,000	0,000						
15	CO-MH779458	A	0,013	0,015	0,013	0,015	0,015	0,015	0,018	0,018	0,018	0,018	0,025	0,008	0,008	0,008					
16	CO-MH779461	AB	0,018	0,020	0,018	0,020	0,018	0,020	0,023	0,023	0,013	0,023	0,030	0,018	0,018	0,018	0,025				
17	CO-MH779462	B	0,028	0,030	0,028	0,030	0,028	0,030	0,030	0,033	0,038	0,033	0,041	0,033	0,033	0,033	0,041	0,036			
18	CO-MH779464	B	0,036	0,038	0,036	0,038	0,036	0,038	0,038	0,041	0,036	0,041	0,048	0,036	0,036	0,036	0,043	0,028	0,008		
19	CO-MH779460	B	0,038	0,041	0,039	0,041	0,038	0,041	0,041	0,043	0,033	0,043	0,051	0,038	0,038	0,038	0,046	0,026	0,010	0,003	
20	CO-MH780915	C	0,129	0,131	0,129	0,131	0,129	0,131	0,131	0,129	0,129	0,134	0,141	0,129	0,129	0,129	0,136	0,127	0,123	0,121	0,119
