# Supplementary material for: The invasive giant African snail Lissachatina fulica as natural intermediate host of Aelurostrongylus abstrusus, Angiostrongylus vasorum, Troglostrongylus brevior, and Crenosoma vulpis in Colombia
Source: PLoS Negl Trop Dis. 2019 Apr 19;13(4):e0007277. doi: 10.1371/journal.pntd.0007277 (PMC6493767; doi:10.1371/journal.pntd.0007277)
Supplement: S1 Text — (DOCX) [file pntd.0007277.s007.docx]

1. Katoh K, Toh H. Improved accuracy of multiple ncRNA alignment by incorporating structural information into a MAFFT-based framework. BMC Bioinformatics 2008; 9:212. doi: 10.1186/1471-2105-9-212.
2. Dereeper A, Audic S, Claverie JM, Blanc G. BLAST-EXPLORER helps you building datasets for phylogenetic analysis. BMC Evol. Biol. 2010; 10:8. doi: 10.1186/1471-2148-10-8.
3. Kumar S, Stecher G, Li M, Knyaz C, Tamura K. MEGA X: Molecular Evolutionary Genetics Analysis across computing platforms. Mol. Biol. Evol. 2018; 35:1547-1549. doi: 10.1093/molbev/msy096.
